# Cyto‐nuclear discordance suggests complex evolutionary history in the cave‐dwelling salamander, *Eurycea lucifuga*

**DOI:** 10.1002/ece3.2212

**Published:** 2016-07-30

**Authors:** Hilary A. Edgington, Colleen M. Ingram, Douglas R. Taylor

**Affiliations:** ^1^ Ontario Institute for Cancer ResearchTorontoONCanada; ^2^ Department of BiologyUniversity of VirginiaCharlottesvilleVirginia; ^3^ Division of Vertebrate BiologyAmerican Museum of Natural HistoryNew York CityNew York

**Keywords:** Cave biology, *Eurycea lucifuga*, habitat colonization, phylogeography, population genetics

## Abstract

Our understanding of the evolutionary history and ecology of cave‐associated species has been driven historically by studies of morphologically adapted cave‐restricted species. Our understanding of the evolutionary history and ecology of nonrestricted cave species, troglophiles, is limited to a few studies, which present differing accounts of troglophiles’ relationship with the cave habitat, and its impact on population dynamics. Here, we used phylogenetics, demographic statistics, and population genetic methods to study lineage divergence, dates of divergence, and population structure in the Cave Salamander, *Eurycea lucifuga,* across its range. In order to perform these analyses, we sampled 233 individuals from 49 populations, using sequence data from three gene loci as well as genotyping data from 19 newly designed microsatellite markers. We find, as in many other species studied in a phylogeographic context, discordance between patterns inferred from mitochondrial relationships and those inferred by nuclear markers indicating a complicated evolutionary history in this species. Our results suggest Pleistocene‐based divergence among three main lineages within *E. lucifuga* corresponding to the western, central, and eastern regions of the range, similar to patterns seen in species separated in multiple refugia during climatic shifts. The conflict between mitochondrial and nuclear patterns is consistent with what we would expect from secondary contact between regional populations following expansion from multiple refugia.

## Introduction

Phylogeographic analysis, examining populations’ evolutionary histories in the context of their spatial distribution, often gives us a better understanding about the formation of lineages and species. Many such studies have examined cave‐dwelling species, which present an interesting and complex look into population divergence given their reliance on such a specific habitat and the adaptive morphological changes that often accompany divergence from surface‐dwelling relatives. The majority of research on cave systems has focused on troglobites (cave‐restricted species), which are characterized by a suite of acquired traits known collectively as troglomorphy. These morphological and physiological traits are thought to act both as a mechanism behind subterranean speciation (Barr et al. 1968; Culver 1982) and as a constraint on dispersal and gene flow between populations in troglobites (Porter 2007). For this reason, troglobites are often characterized by extremely limited geographic range (Culver and Holsinger 1992; Christman et al. 2005); for example, the majority of US troglobites are endemic to a single county (Culver et al. 2000).

Although a great deal of attention is paid to troglobitic species, there are other classes of cave‐dwelling species. Of particular interest, here is the class of cave‐dwellers known as troglophiles. These species maintain permanent cave populations, but vary in their restrictedness to the cave habitat (Sket 2008). For some taxa, not being restricted to the cave habitat allows greater dispersal in troglophilic populations compared to troglobitic populations. Caccone (1985) studied several species of cave arthropods with varying cave restrictedness and found that while the amount of gene flow between populations and population structure varied tremendously among all species, and were highly dependent on both the characteristics of the cave and surface environments as well as innate characteristics of the species, troglobites tended to have lower levels of gene flow among caves than troglophiles (Caccone 1985). In contrast, populations of the troglophilic isopod *Androniscus dentiger* exhibited surprisingly high differentiation with no detectable interpopulation gene flow (Gentile and Sbordoni 1998). Similarly, very low levels of gene flow and high population structure have been described in troglophilic Sardinian cave salamanders (genus *Hydromantes*), likely due to the inhospitable surface environments that discourage dispersal outside of caves (Chiari et al. 2012). These studies suggest that, depending on environmental and species characteristics, troglophiles may experience restriction to caves in the same way that troglobites do despite lacking many of the morphological characteristics that make troglobites better suited for the subterranean environment. However, the relative lack of attention paid to troglophilic species prevents us from understanding how inhabiting the cave environment impacts the population and demographic dynamics of a species where cave association is not mandated by morphological restrictions, as it is in troglobitic species.

### Study system

**Figure 1:**
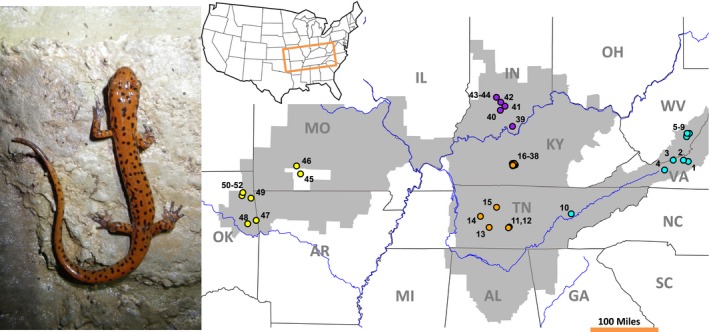
Depiction of the range of *Eurycea lucifuga* (shown in gray) and collection locations (circles). Colors of population markers are reflective of the major regional lineages detected in population genetic analyses. Sampling information for each locality can be found in Table 1 and Table S1, Supporting Information.

We studied the phylogeographic history and population dynamics of a troglophilic salamander in the genus *Eurycea* (Plethodontidae). *Eurycea* is particularly notable for its abundance of troglobitic species. Although the ancestors in this genus were surface‐dwelling, troglobitism evolved independently at least five times within the genus (Bonett et al. 2014). *Eurycea lucifuga* is a troglophilic salamander commonly found in limestone caves throughout the Southeast and Midwest United States (Fig. 1). *Eurycea lucifuga* has a multistage life cycle, in which females lay eggs deep underground in aquatic pools. The eggs hatch into larvae, which are restricted to these subterranean pools until they fully metamorphose into brightly colored adults (Fig. 1; Petranka 1998). Adult *E. lucifuga* generally live within caves close to the entrance, although individuals are often found deep within caves at distances beyond 1000 m from the entrance (H. A. Edgington, pers. observ.). However, there are anecdotal reports of adult *E. lucifuga* observed in the forest matrix surrounding caves (Hutchison 1958; Petranka 1998).

Despite the frequency with which *E. lucifuga* is found across its wide range and its occupancy of a well‐studied habitat, very little is known about its evolutionary history in the context of its current range. Understanding these important attributes is a critical component of examining other aspects of its ecology and life history. We investigated the phylogeography of *E. lucifuga* using phylogenetic, population genetic, and demographic statistical methods in order to better understand its evolutionary history. First, we used a tree‐building approach to reconstruct the phylogeographic history of populations across the range of *E. lucifuga* and estimated the timing of divergence between populations in different geographic regions. Second, we used a classical population genetics approach to assess how genetic variation is structured across the range, and to infer a reconstruction of historic lineage splitting.

Published information about the evolutionary history of other salamanders and other cave‐dwelling species reveals some general phylogeographic patterns that shape our expectations. Many North American salamander species experienced ancient divergence from sister taxa, on the order of millions of years (Bonett and Chippindale 2004; Kozak et al. 2006; Reilly and Wake 2014; Folt et al. 2016), but in most species, intraspecific divergences reflect the impacts of climatic fluctuations throughout the Pleistocene (approximately 2.5 Ma–10 Ka; Church et al. 2003; Herman and Bouzat 2015; Newman and Austin 2015). The evolutionary history of troglophilic salamanders tends to reflect these same patterns, for example, *Hydromantes strinatii*, a native to Southern Europe, experienced divergence among major lineages during the Pleistocene (Cimmaruta et al. 2015). The ability to persist both within and outside of caves allowed populations to remain stable during climatic changes following lineage divergence, resulting in high genetic diversity within populations, with isolation due to geographic barriers resulting in high geographic substructuring within major lineages (Chiari et al. 2012; Cimmaruta et al. 2015). The *Gyrinophilus porphyriticus* complex exhibits a similar pattern, with major lineages diverging before the Pleistocene, followed by isolation among geographic regions throughout the Pleistocene, resulting in geographic substructuring correlated with historic drainage basin patterns (Kuchta et al. 2016). Exceptionally high local genetic structure is often a signature of both troglophilic and troglobitic species, including *Astyanax fasciatus* (Strecker et al. 2004), *Pseudouroctonus reddelli* (Bryson et al. 2014), *Amblyopsis spelaea* (Niemiller et al. 2013), Western Australian amphipods (Finston et al. 2007), and *Nesticus barri* (Snowman et al. 2010), due to barriers between caves. However, Caccone (1985) determined that dispersal ability in cave‐dwelling species is variable and largely dependent on their ecological requirements.

Previous studies focusing on speciation among a broad group of salamander taxa have inferred that speciation between *E. lucifuga* and its sister taxon *Eurycea longicauda* occurred approximately 10 Ma (Kozak et al. 2009; Bonett et al. 2014; Martin et al. 2015), and ancestral habitat reconstruction provides some evidence for a dispersal event from the eastern United States to the West approximately 4 Ma (Martin et al. 2015). Because these studies only included one or two samples in their analyses, broader sampling and more thorough analyses are needed to better understand the evolutionary history of *E. lucifuga*.

## Materials and Methods

### Data collection

Between 2012 and 2013, we collected tail clips from one to 11 individuals from populations of *E. lucifuga* throughout its range (Fig. 1; Table 1; list of all individuals found in Table S1, Supporting Information). A population is defined here as a group of individuals collected from inside single cave entrance. This definition can be problematic in cases where cave entrances are part of a larger cave system; however, to the best of our knowledge the majority of cave entrances in this study are not connected. Animals were released at their capture site following collection.

**Table 1:** A summary of sampling per population. Numbers correspond to marker labels in Figure 1

Designation	State	Cave name	Samples	Region
3	VA	Blankenship Blowhole	6	Eastern
4	VA	Byrd's Water	10	Eastern
2	VA	Smokehole	6	Eastern
1	VA	Tawney's	3	Eastern
5	WV	Borehole	2	Eastern
6	WV	Buckeye Creek	1	Eastern
7	WV	Higginbotham	1	Eastern
8	WV	Mann	3	Eastern
9	WV	Spring	1	Eastern
10	TN	Lost Puddle	1	Eastern
39	IN	Bankley/Fairground	9	North‐central
42	IN	Donnehue	5	North‐central
41	IN	Roberts	4	North‐central
43	IN	Sullivan	6	North‐central
44	IN	Robinson Ladder	4	North‐central
40	IN	The Lost River	6	North‐central
16	KY	Adwell	1	South‐central
17	KY	Big Hollow Cave	7	South‐central
18	KY	Black Rock	1	South‐central
19	KY	C2	7	South‐central
20	KY	Cadaverous Cave	5	South‐central
21	KY	Crystal Cave	2	South‐central
22	KY	Falling Tree Cave	6	South‐central
23	KY	Great Onyx Cave	11	South‐central
24	KY	Hickory Cabin Cave	6	South‐central
25	KY	Left Eye Cave	1	South‐central
26	KY	Little Beauty	2	South‐central
27	KY	Mammoth Cave	2	South‐central
28	KY	Natural Bridge Cave	1	South‐central
29	KY	New Discovery Entrance	11	South‐central
30	KY	Pagoda	3	South‐central
31	KY	Phil Cave	4	South‐central
32	KY	Silent Spring	6	South‐central
33	KY	Stan	2	South‐central
34	KY	Sturgeon Cave	1	South‐central
35	KY	Unknown Cave (Austin Ent.)	2	South‐central
36	KY	Violet City Entrance	6	South‐central
37	KY	White Cave	2	South‐central
38	KY	YMCA	8	South‐central
11	TN	Crews	4	South‐central
12	TN	Eoff	6	South‐central
15	TN	Gillespie	4	South‐central
14	TN	Mull Prowell	6	South‐central
13	TN	Pompie	3	South‐central
44	MO	Bull Creek	9	Western
45	MO	Crighton Spring	5	Western
48	OK	Blue Moon	2	Western
49	OK	Iron Gate	3	Western
50	OK	Jail	9	Western
51	OK	January‐Stansbury	7	Western
47	OK	Survivalist	2	Western
46	OK	Third	4	Western

Tissue samples were stored in 95% ethanol at room temperature at the University of Virginia (Charlottesville, VA). We extracted total genomic DNA from each sample using either a phenol–chloroform method (Sambrook et al. 1989) or a 5–10% slurry of Chelex with an incubation time of 180 min at 95°C. We then amplified three gene fragments commonly used in salamander systematics using polymerase chain reaction (PCR): two mitochondrial loci, NADH dehydrogenase 2 (ND2) and cytochrome *b* (cyt*b*), and one nuclear locus, proopiomelanocortin (POMC). Primer information and thermocycling conditions are shown in Table S2 (Supporting Information). We cleaned the PCR products using ExoSAP‐IT (Affymatrix, Santa Clara, CA) and then sequenced both directions using Sanger sequencing on an ABI 3730*xl* at GENEWIZ (South Plainfield, NJ). We used the software program Geneious v6.1.6 (Biomatters, Aukland, New Zealand) to edit and build contigs of the resulting sequence. For each locus, sequences passing a quality threshold (>75% of sites designated high quality) were aligned using the Geneious alignment option. Our resulting alignments were unambiguous, and there were no gaps in the sequence. We included sequence data for each locus from *E. longicauda* (this study) and *Pseudotriton ruber* (cyt*b*: JQ920615.1, ND2: JQ920799.1, POMC: EU275854.1). All sequences are deposited in GenBank (*cytb*: KX148758–KX148830, ND2: KX148831–KX148903, POMC: KX148904–KX148976). We used Xia's Index of Sequence Saturation in DAMBE v5.2 (Xia et al. 2003; Xia and Lemey 2009) to test whether each locus was phylogenetically informative.

### Microsatellite development and genotyping

In order to isolate species‐specific microsatellite markers, tail clips were taken from two individuals collected from different localities in Southwestern Virginia in Spring 2014 and immediately preserved in RNAlater (Life Technologies, Carlsbad, CA). Whole sample RNA was extracted using a Qiagen RNEasy Mini Kit (Qiagen, Valencia, CA) following manufacturer's suggested protocols and processed in the Genomic Core Facility at the University of Virginia's Department of Biology for the construction of individual paired‐end, non‐normalized cDNA libraries using a NEBNextmRNA library Prep Master Mix Set (New England Biolab, Ipswich, MA). Individual libraries were given barcodes and pooled, and sent to Genewiz (GENEWIZ, South Plainfield, NJ) for sequencing on the Illumina HiSeq 2500 using a PE 2 × 100 bp format. Resulting sequences from each sample were assembled using the program Trinity v.11‐10‐2013 (Grabherr et al. 2011), and a summary of the sequencing results for each library can be found in Table S3 (Supporting Information). A custom BLAST search was used to locate regions of the mitochondrial genes cyt*b* and ND2 sequenced previously from *E. lucifuga* in order to confirm the identity of the samples. Putative microsatellite loci were identified in each group of transcripts using the program msatcommander v. 1.0.8 (Faircloth 2008), specifying a search for repeats of trinucleotides or greater with at least five repeats. We used BLAST to confirm the presence of primer sequences in both samples, and discarded primers that were not present in both group of transcripts.

Following the method of Schuelke (2000), we screened each primer set for amplification and polymorphism using m13 fluorescently labeled tags. Each test was performed using eight samples collected from across the species range, and confirmational sequencing was performed using fragment analysis on an ABI 3130 Sequencer (Life Technologies). Primer pairs that amplified in all eight test samples and were polymorphic were produced with fluorescent tags incorporated into each forward primer and multiplexed. Thermocycler conditions used throughout the screening process are as follows: 94°C for 15 min, 40 cycles of 94°C for 30 sec, 60°C for 1:30 min, and 72°C for 1:30 min, and finally 72°C for 10 min. After identifying putative microsatellite loci and using BLAST to validate them, we had 220 markers to screen. Twenty‐four of these amplified in all test individuals and were polymorphic (>2 alleles), and these were multiplexed into six multiplexes, each containing four loci Table S4 (Supporting Information).

Loci were amplified in multiplexed reactions using PCR, and products were sent to the DNA Analysis Facility on Science Hill (Yale University, New Haven, CT) for fragment analysis using a 3730*xl* 96‐Capillary Genetic Analyzer, with the DS‐33 dye set. Finally, we used GeneMarker v2.4 (SoftGenetics, State College, PA) to call alleles at each marker locus for each individual.

### Phylogenetic analyses

We analyzed the sequence data from cyt*b*, ND2, and POMC individually and as a combined, partitioned dataset under maximum likelihood (ML) and Bayesian inference (BI) using RAxML 7.2.6 (Stamatakis 2006) and MrBayes 3.1.2 (Ronquist et al. 2012). Individuals with missing data were excluded from any reconstructions where the loci were included in a combined analysis. Appropriate models of sequence evolution were inferred for each locus using the Akaike information criterion, Bayesian information criterion, and Decision Theory Performance‐Based Selection (DT) in the program jModelTest v2.1.4 (Table S5, Supporting Information; Posada 2008). If these methods disagreed, we used the model chosen by two of the three. When specific models were unavailable, we underparameterized by choosing a slightly simpler model. The final models we used for further analysis were HKY+I+G (cyt*b*), HKY+I (ND2), and HKY (POMC). We performed ML phylogenetic analysis using the ML + thorough bootstrap analysis option with two independent runs and 1000 nonparametric bootstraps. We used Bayes Factors implemented in MrBayes to compare the heuristic mean likelihood of two BI analyses, in which one model specified partitioning among codons and one model specified a simpler partitioning scheme with only partitioning among the gene loci. This comparison indicated that the simpler partitioning model was more successful at predicting the data (individual codon partitioning: −lnL = −6000.91; combined codon partitioning: −lnL = −5788.31; Kass and Raftery 1995). With the correct partitioning model and appropriate model of substitution, and linking the topology parameter for the two mitochondrial loci, each MrBayes analysis was run for 50M generations and trees were sampled every 2500 generations, with three heated chains and one unheated chain. We analyzed the Bayesian results for convergence as well as comparing individual searches and ensuring ESS values were greater than 200 using Tracer v1.6.0 (Drummond et al. 2012). We used TreeAnnotator v1.8.1 (Drummond et al. 2012) to sample the trees and infer a single Maximum Clade Credibility tree for each analysis, discarding the first 20% of the generations as a burn‐in. We visualized trees using the program FigTree v 1.4.2 (Drummond et al. 2012), including ML bootstrapping and BI posterior probability information at each well‐supported major clade on the resulting BI tree.

We used *BEAST v1.8.1 (Drummond et al. 2012) to infer a species tree wherein “species” were representative of individual populations (Ex: Smoke Cave, Tawney's Cave, Byrd's Water were considered individual “species”). This assignment reflects our interest in divergence among populations at a regional scale. All three loci were included in this analysis as separate partitions, with all parameters except for the tree parameter unlinked, and with the two mitochondrial loci trees linked. As we are mainly interested in intraspecific divergence estimates, we constrained all *E. lucifuga* individuals to be monophyletic, with *E. longicauda* and *P. ruber* as outgroups, and focused on divergence estimates within *E. lucifuga*. We used a stepping‐stone analysis comparing the use of a relaxed molecular clock with a strict clock model implemented in MrBayes for 50 steps, each with 19,500 generations, which indicated that the strict clock model had a higher mean marginal likelihood (strict marginal likelihood, −lnL = −6288.50, relaxed marginal likelihood, −lnL = −6334.81). Accordingly, we used previously published rates to calibrate the clock for *cytb* and ND2 (0.0062 substitutions/my and 0.0037 substitutions/my, respectively; Mueller 2006) and estimated the rates of POMC with reference to the mitochondrial rates. We ran the two independent analyses for 500M generations, and sampling trees for every 5000 generations under a Yule model with piecewise linear population size and a constant root. We used Tracer v1.6.0 (Drummond et al. 2012) to compare the independent runs, ensure that all ESS values were >200, and test for convergence. We discarded the first 20% of the resulting trees as a burn‐in, combined the independent runs with LogCombiner v1.8.1 (Drummond et al. 2012), and inferred a single tree with TreeAnnotator v1.8.1 (Drummond et al. 2012). This tree was visualized using Figtree v1.4.2 (Drummond et al. 2012). Additionally, median‐joining networks (Bandelt et al. 1999) were inferred for each locus separately using the software package PopART v1.7 (Allan Wilson Centre, http://popart.otago.ac.nz).

### Demographic statistics

To test for departures from neutrality in the three sequenced loci, we estimated Tajima's *D*, which compares segregating sites with average pairwise nucleotide divergence (Tajima 1989), Fu's and Li's *F*, which compares haplotype variation compared with average pairwise nucleotide divergence (Fu and Li 1993), and Ramos‐Onsin's and Rozas's *R* ^2^, which compares singleton mutations with average pairwise nucleotide divergence (Ramos‐Onsins and Rozas 2002). Significantly negative estimates of any of these parameters are indicative of either recent population expansion or purifying selection, while significantly positive estimates may indicate a recent bottleneck or overdominant selection. We used the software program DnaSP v5 (Librado and Rozas 2009) to produce these estimations in the entire population and within the eastern, north‐central, south‐central, and western regions.

### Population genetic analyses

Population genetic analyses were performed using the microsatellite genotype data. We performed a cluster analysis using STRUCTURE v2.3.4 (Pritchard et al. 2000). We used the admixture model, with independent allele frequencies. Following the methods of Coulon et al. (2008), we first ran the analysis with *K* = 2, and then analyzed each partition separately using a range (*K* = 1–10, for a total of up to 20 potential *K* groups). The two iterative runs for each *K* scenario included 20,000 steps as a burn‐in, followed by 200,000 more. The most appropriate number of clusters was determined using the Evanno method (Evanno et al. 2005) in Structure Harvester web v0.6.94 (Earl 2012). We then visualized the clustering in distruct v1.1 (Rosenberg 2003; Jakobsson and Rosenberg 2007). To test whether the pattern of variation could be explained by isolation by distance, we used Genepop v4.2 (Raymond and Rousset 1995; Rousset 2008) to examine the correlation between genetic and geographic distances with Mantel tests. We performed a separate analysis within each of the main clusters found by STRUCTURE using 1000 permutations of the Mantel tests.

**Figure 2:**
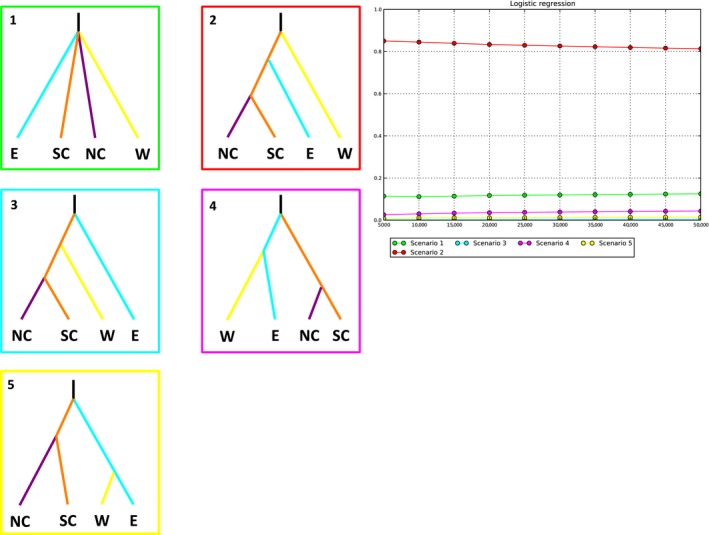
The five scenarios tested in the DIYABC model, depicting hypothetical relationships among the eastern, north‐central, south‐central, and western populations, with branch colors corresponding to regions in Figure 1. On the right, the results of a logistical model comparing the posterior probability of each scenario with the number of simulations used to calculate it; colors reflect each evolutionary scenario.

We examined genetic diversity within each region by comparing estimates of heterozygosity and inbreeding coefficients directly. To estimate how variation is partitioned among‐ and within‐groups and populations, and to estimate Wright's *F* statistics, we used analysis of molecular variance (AMOVA) in the program GenoDive v2.0b27 (Meirmans and Van Tienderen 2004). We used a principal component analysis on allele frequencies among populations in order to visualize clustering in the microsatellite data. This was performed in GenoDive (Meirmans and Van Tienderen 2004) using the covariance matrix method. The resulting axes of variance were tested for significance using 99 permutations. We tested for significant differences in the variation explained by the first two principal components using ANOVA followed by a Tukey test in the R packages *aov()* and *TukeyHSD()*. Finally, to compare divergence at a regional scale using microsatellite data with the results of the gene‐based phylogenetic methods, we used approximate Bayesian computation (ABC) in the program DIYABC v2.0 (Cornuet et al. 2014) to infer the likelihood of different divergence scenarios. We tested five scenarios, that included four clusters consistently recovered by the *BEAST and STRUCTURE analyses, representing the East, West, and splitting the central cluster into north‐central and south‐central. The five scenarios included one with equal ages for each major clade and four additional scenarios designating each major region as the outgroup to the other clades (Fig. 2). We assumed the microsatellites were evolving under a Generalized Stepwise Mutation model, using the default priors for each analysis, and ran the simulation for 5,000,000 iterations. We performed model selection on our simulation results, using both a direct estimation of the most likely scenario with 500 iterations and a logistical regression using 4000 iterations. Model checking was used to compare axes of variation within the summary statistics produced from the simulation to those predicted by the posterior predicted distribution of the best‐fitting model. Upon obtaining DIYABC's estimates for the timing of divergences among lineages, we multiplied the generation times by estimates of the age of sexual maturity in *E. lucifuga* to infer a range of ages in years. Sexual maturity in this species is reached at approximately 2.5–4 years (Petranka 1998), so we multiplied each time estimate by 2.5 and by 4. Given the variability in first reproduction in this species, as well as the error in the timing estimates, however, the ages of divergence are only approximate.

## Results

Fragments of cyt*b* (734 bp), ND2 (565 bp), and POMC (409 bp) (1708 bp total) from 84 individuals from 27 populations (indicated in Table S1) were included in our phylogenetic reconstructions and demographic statistical analyses. None of our loci exhibited significant saturation (cyt*b*:* T* = 49.061, df = 463, *P *<* *0.0001; ND2: *T* = 62.6957, df = 549, *P *<* *0.0001; POMC: *T *=* *122.5041, df = 465, *P *<* *0.0001), indicating that our sequence data contain useful phylogenetic information.

Nineteen microsatellite loci were successfully amplified in 233 samples of *Eurycea lucifuga* collected from 49 populations across its range (indicated in Table S1; Supporting Information); 5 loci exhibited inconsistent amplification and were dropped from further analyses. Allele number at each locus (*k*) ranged from 2–7 (Table S4; Supporting Information). Observed and expected heterozygosity (*H*_o_ and *H*_E_) were estimated using GenoDive v.2.0b27 (Meirmans and Van Tienderen 2004), and are shown in Table S4 (Supporting Information). No loci significantly deviated from HWE within populations (Table S6; Supporting Information). All analyses were run using 999 permutations.

### Phylogeographic analyses (DNA sequence data)

**Figure 3:**
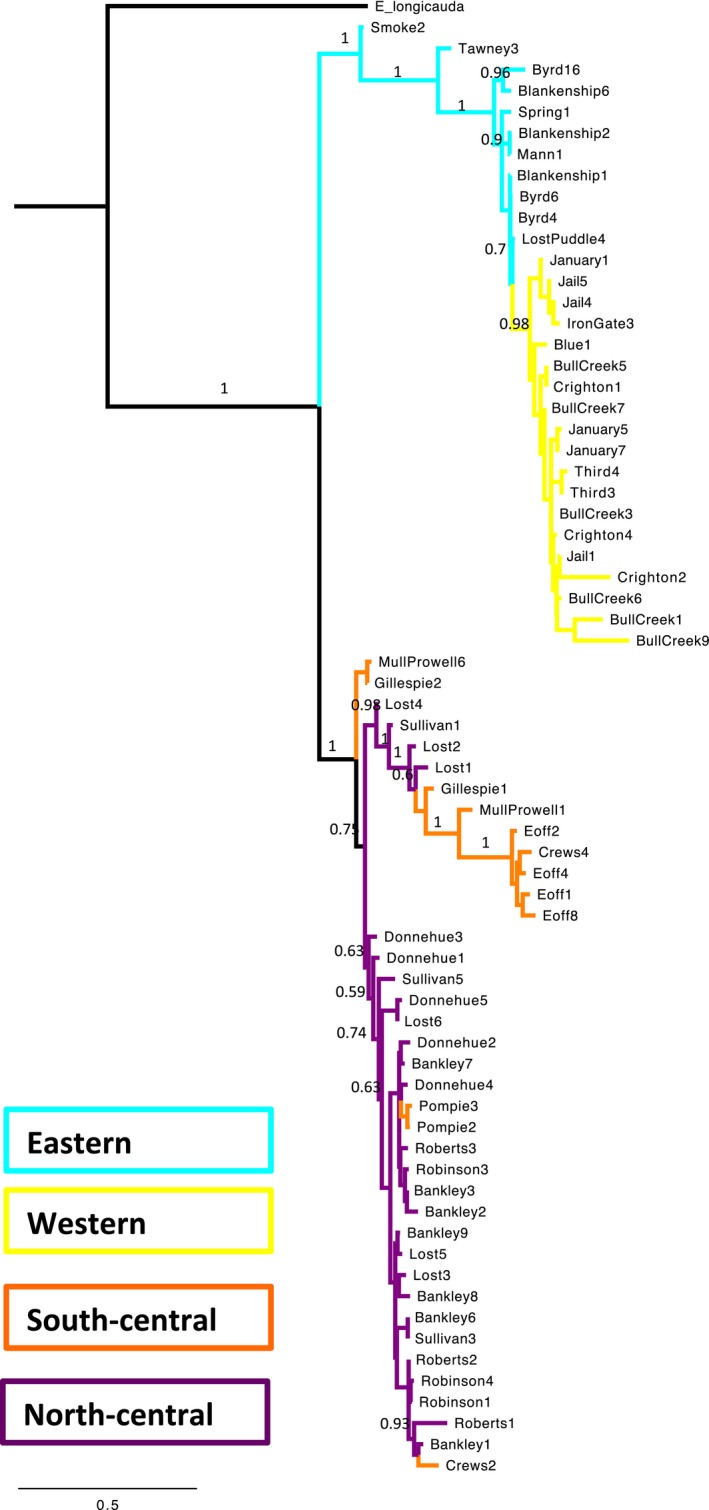
Bayesian phylogeny inferred from the concatenated sequence data of cyt*b*, ND2 and POMC using MrBayes. *Pseudotriton ruber* was included as an outgroup, but is not shown here for ease of visualization. Posterior support values above 0.5 are shown above, and maximum‐likelihood bootstraps above 50 are shown below branches. Colors correspond to the regions from which samples were collected, as seen in Figure 1.

The ML and Bayesian concatenated trees both recover two well‐supported major clades organized by geographic region; the topologies are very similar with only minor rearrangements within major clades, and therefore, we present here the Bayesian tree with ML bootstrap values shown (Fig. 3). The central clade contains populations in western Tennessee and Indiana, and the eastern/western clade contains the populations within Virginia, West Virginia, eastern Tennessee, Oklahoma, and Missouri. The western populations form a monophyletic clade within the eastern populations. Haplotypes were shared among populations within the major geographic regions; however, support at the tips was very low. Relationships among the clades differ from simple geographic expectations: the western and eastern regions are more closely related to each other than either is to the central clade. Gene trees of individual loci (Figures S1–3, Supporting Information) generally reflect these relationships, although the nuclear locus POMC did not have a large influence on the topology, and as a result, the combined trees generally represent the mitochondrial history of the species.

**Figure 4:**
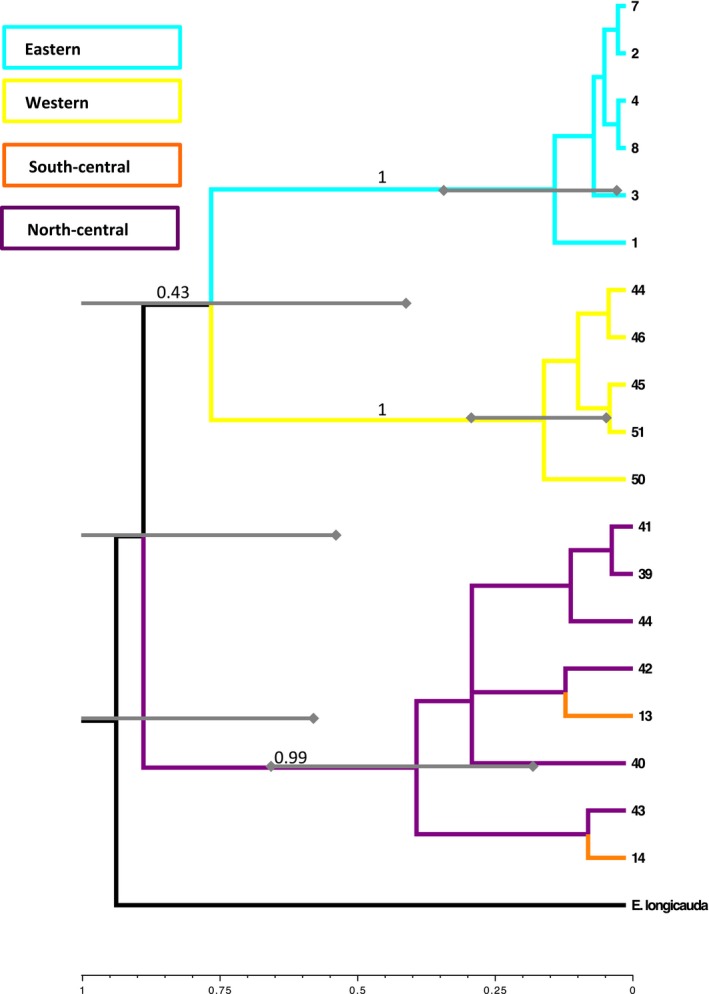
A species tree produced in *BEAST with a strict molecular clock, indicating divergence of the major lineages during the mid‐late Pleistocene. Bars on major splits represent the 95% CI of the age of that node, and posterior probabilities of major lineage divergences are represented on branch labels. Relationships at the tips were not well supported. Taxon labels reflect the number designation given to each population in Figure 1 and Table 1.

All parameters of the *BEAST analysis, when viewed in Tracer, had ESS values much higher than 200, and the independent runs reached convergence. The species tree produced in *BEAST (Fig. 4) is very similar to those produced using traditional concatenation‐based methods, with the central region forming an outgroup to the eastern and western regions. However, in the species tree, the eastern and western regions are reciprocally monophyletic, whereas the concatenation trees feature the inclusion of the western clade within the eastern populations. Support for all three major clades is high in the species tree; however, support for the internode splitting the eastern and western clades is extremely low (0.43) indicating that there is significant ambiguity in this topology. Examination of the gene trees produced by running each locus individually in MrBayes and RaxML and the gene trees produced in *BEAST shows the majority of the signal in the concatenated and species trees is due to significant clustering from mitochondrial data. As in the concatenated trees, the topology of the species trees is very similar to the *cytb* and ND2 gene trees, while the POMC gene trees exhibit very little population or regional clustering, indicating that the species tree is largely representative of the mitochondrial history of this species.

**Figure 5:**
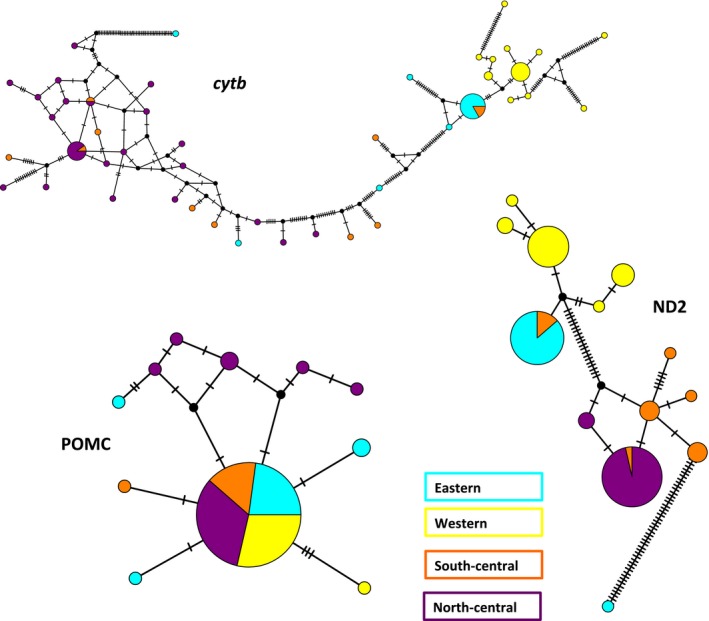
Median‐joining networks for each gene locus generally reflect the relationships inferred by the tree‐building methods; the POMC network indicates that much of the clustering we see in the trees is reflective of the mitochondrial history of this species. Colors correspond to the regional lineage colors seen in Figure 1.

Diversity is extremely low in the nuclear locus, POMC, and highest in the mitochondrial locus *cytb*. When studying each sequenced locus separately as a haplotype network, it is clear that sharing of haplotypes among regions is common most frequently between the south‐central region and either the eastern or western regions. Both mitochondrial networks indicate that sharing of haplotypes among regions is common even between the eastern and western regions (Fig. 5).

### Divergence dating (DNA sequence data)

The chronogram inferred by *BEAST (Fig. 4) dates the divergence between the central and eastern/western clades at approximately 0.88 Ma, and the divergence between the eastern and western clades at approximately 0.77 Ma. Diversification within the major clades is estimated to have occurred more recently than approximately 0.39 Ma (central populations), 0.16 Ma (western populations), and 0.14 Ma (eastern populations). However, these dates should be interpreted with caution due to the ambiguity in the relationships among clades, which could impact age estimations.

Tests of departures from neutrality, which could indicate population expansion or contraction, reveal that recent expansions in the western and eastern regions are supported by significantly negative values of Tajima's *D* and Fu and Li's *F* statistics, while a significantly negative value of Ramos‐Onsins and Rozas's *R* ^*2*^ test adds further support to recent expansion in the West. Neither the north‐central nor the south‐central region shows any evidence of departures from neutrality (Table 2).

**Table 2:** Measures of heterozygosity and demographic statistics for each of the major lineages as well as all sampled individuals. Significant departures from neutrality (at *P* < 0.05) for the demographic statistics are in bold. Heterozygosity was significantly greater in the central regions than the East and West (shown)

	All samples	East	West	North‐central	South‐central		
Pi	0.032	0.014	0.008	0.006	0.021		
Fu and Li's *F*	−**2.522**	−**3.123**	−**2.655**	−1.657	−0.332		
Tajima's *D*	−0.077	−**2.286**	−**1.968**	−1.424	−0.213		
*R* ^2^	0.094	0.132	**0.126**	0.08	0.152		
						Osx	*P*
*H*_o_	0.184	0.114	0.165	0.183	0.217	0.149	**0.003**
*H*_s_	0.224	0.163	0.163	0.226	0.269	0.18	**0.003**
*G*_is_	0.178	0.303	−14	0.192	0.195	0.459	**0.012**

### Population genetic analyses (Microsatellite data)

**Figure 6:**
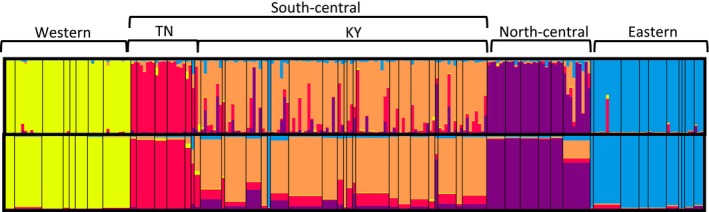
The geographic clustering of diversity, estimated using STRUCTURE, indicates four major clades across the range of *Eurycea lucifuga*. While the eastern and western clades are distinct from each other entirely, they both contribute to the diversity found within the two central clades, indicating either asymmetric gene flow or past expansion from the central region.

**Figure 7:**
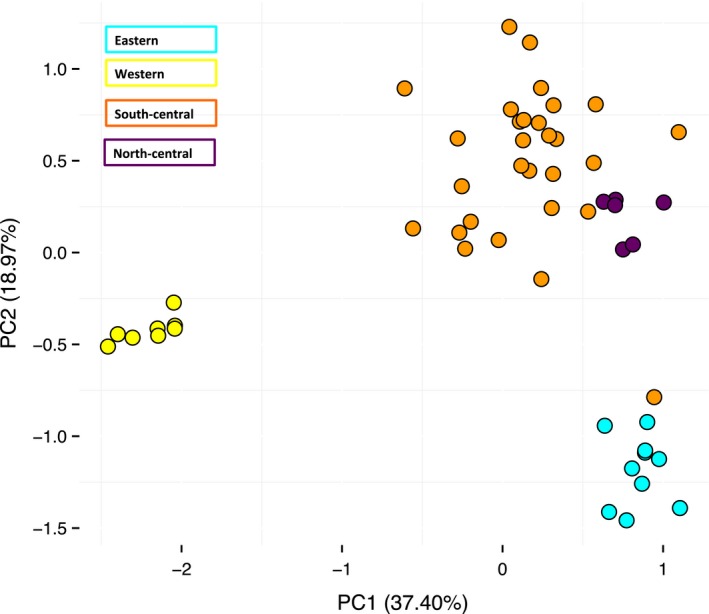
A visualization of the Principal Component Analysis performed on allelic diversity among populations, where color corresponds to region as in Figure 1. Results of ANOVA and subsequent post hoc tests indicate significant differences between all regions with the exception of the eastern and north‐central regions in PC1, and the north‐central and south‐central regions in PC2 (Table 4).

The STRUCTURE analysis indicates that the model with the highest likelihood is *K* = 5 (likelihood scores of the two partitions shown in Table S7, Supporting Information). Assignment to these genetic clusters partitions the samples into clear geographic groups: western, north‐central (Indiana), south‐central (Kentucky), south‐central (Tennessee), and eastern populations (Fig. 6). Allele sharing is common particularly between the two south‐central regions, but also among the south‐central and north‐central populations, and to a lesser extent among the south‐central and eastern populations. We found no significant evidence of isolation by distance within the western or eastern populations; however, correlations between genetic and geographic distances were significant among populations in the north‐central region (*r *=* *0.05, *P *=* *0.023) and in the south‐central region (*r *=* *0.07, *P *=* *0.043). When compared directly, the central regions contain significantly more heterozygosity than the western or eastern regions (Table 2), so there may have been greater power to detect subtle patterns of isolation by distance. AMOVA results indicate significant population structure among sampling localities within the eastern, central and western regions (*F*_SC_ = 0.093, *P* = 0.001) and strong population genetic divergence among regions (*F*_CT_ = 0.331, *P* = 0.001). Thirty‐three percent of genetic variance is partitioned among regions and 6% among localities within each region. A substantial amount of variation is partitioned among individuals within localities (13%, *F*_IS_ = 0.208, *P* = 0.001) (Table 3). Results of the PCA also indicate strong clustering among regions (Fig. 7). The first two principal components accounted for 37.4% (*P* = 0.03) and 18.97% (*P* = 0.04) of the total variance, respectively, and exhibited significant clustering among populations. There were significant differences in the variation in PC1 among all regions except between the eastern and north‐central regions, and significant differences in variation in PC2 between all regions except for between the south‐central and north‐central regions (Table 4). Comparisons of the different divergence scenarios in DIYABC indicated that the most likely scenario includes a primary splitting of the western populations, followed by a subsequent divergence between the central and eastern populations (Table 5, Fig. 2). Population size estimates indicate that effective population size in the north‐central and south‐central regions is greater than that in the eastern and western regions by almost an order of magnitude (Table S8, Supporting Information). After applying current knowledge of the age at sexual maturity in *E. lucifuga* to the estimates of time since divergence in generations among lineages, results suggest that divergence between the north‐ and south‐central regions occurred approximately 1772–2836 years ago, the split between the eastern and two central clades occurred 2925–4680 years ago, and western divergence occurred 9650–15,440 years ago (Figure S4, Table S8, Supporting Information). However, it has been found that microsatellite data tends to underestimate timing of events, specifically ancient events (Cornuet et al. 2010), and so, we treat these results with caution.

**Table 3:** AMOVAs across the range as well as within each region indicate that the majority of genetic variation is partitioned among regions, and that regions differ widely in the amount of population structure they exhibit. Significant results are shown in bold

Source	Nested in	%var	*F*‐stat	*F*‐value	Std. dev.	CI 2.5%	CI 97.5%	*P*‐value
Range‐wide								
Within individual	–	0.613	*R*_it_	0.387	0.26	0.164	0.705	–
Among individual	Population	0.014	*R*_is_	0.022	0.233	−0.111	0.435	0.279
Among population	Region	0.038	*R*_sc_	0.057	0.03	0.009	0.074	**0.001**
Among region	–	0.335	*R*_ct_	0.335	0.188	0.16	0.593	**0.001**
Western								
Within individual	–	1.184	*R*_it_	−0.184	0.487	−0.333	0.609	–
Among individual	Population	−0.201	*R*_is_	−0.204	0.511	−0.368	0.619	0.942
Among population	–	0.017	*R*_st_	0.017	0.023	−0.043	0.06	0.249
South‐central								
Within individual	–	0.829	*R*_it_	0.171	0.094	0.056	0.432	–
Among individual	Population	0.087	*R*_is_	0.095	0.144	−0.023	0.427	**0.039**
Among population	–	0.084	*R*_st_	0.084	0.06	0.004	0.127	**0.001**
North‐central								
Within individual	–	1.037	*R*_it_	−0.037	0.311	−0.216	0.454	–
Among individual	Population	−0.092	*R*_is_	−0.098	0.337	−0.292	0.421	0.842
Among population	–	0.055	*R*_st_	0.055	0.018	−0.001	0.1	**0.046**
Eastern								
Within individual	–	0.859	*R*_it_	0.141	0.289	0.02	0.679	–
Among individual	Population	0.147	*R*_is_	0.146	0.264	0.044	0.681	0.123
Among population	–	−0.006	*R*_st_	−0.006	0.042	−0.036	0.057	0.534

**Table 4:** Principal Component analysis of microsatellite allele diversity demonstrates clustering within regions. Variance within regions is significantly different, with the exception of clustering between the eastern and north‐central regions, and between the south‐central and north‐central regions. Significant results of Tukey tests between regions are shown in bold

Axis	Eigenvalue	% Variance	Cumulative	*G*′_st_(Nei)	*P*
PC1	1.052	37.401	37.401	0.112	0.03
PC2	0.534	18.973	56.373	0.057	0.02

Comparison of PC1 among regions					
*F*	df	Mean square	*P*		
179.06	3	16.7075	<0.0001		

	Eastern	North‐central	South‐central		
North‐central	−0.084				
South‐central	−**0.696**	−**0.612**			
Western	**3.049**	−**2.965**	−**2.353**		

Comparison of PC2 among regions					
*F*	df	Mean square	*P*		
70.251	3	7.5039	<0.0001		

	Eastern	North‐central	South‐central		
North‐central	**1.378**				
South‐central	**1.67**	0.292			
Western	**0.764**	−**0.614**	−**0.906**		

**Table 5:** Posterior probabilities (PP) and 95% confidence intervals (CI) for the five‐scenario comparison using DIYABC. Scenarios are detailed in Figure 2. Highest PP (Scenario 2) is shown in bold

	Posterior probability	95% CI
Scenario 1	0.1252	0.0377, 0.2127
Scenario 2	**0.8136**	0.7946, 0.8326
Scenario 3	0.0035	0.0000, 0.0869
Scenario 4	0.0432	0.0000, 0.1235
Scenario 5	0.0145	0.0000, 0.0974

## Discussion

We found substantial discordance between mitochondrial and nuclear signal, with contrasting inferences of the evolutionary history of *E. lucifuga*. Results of the phylogenetic analyses and demographic statistics using mitochondrial‐driven sequence data suggest that within *E. lucifuga,* there is deep divergence between three major lineages: eastern populations, central populations, and western populations. Relationships among major lineages suggest that there was an initial divergence between the central clade and the eastern/western clade, followed by a later split between the eastern and western clades. This evolutionary scenario is reflected in all tree‐building methods, as well as in haplotype networks. However, support for a close relationship between the eastern and western clades was very low in the species tree recovered by *BEAST, indicating ambiguity in the topology. We also see shared haplotypes among populations within regions and among regions in the concatenation‐based tree‐building methods as well as haplotype networks, which suggests that either there is persistent gene flow among populations or there has been incomplete lineage sorting among regions.

While a closer relationship between eastern and western clades than between either eastern or western and central clades appears counterintuitive, this pattern has been seen in other North American salamanders. For example, the same pattern of divergence is seen among major lineages in the Spotted Salamander, *Ambystoma maculatum* (Phillips 1994; Zamudio and Savage 2003), which was hypothesized to be the result of expansions from two glacial refugia: one lineage expanded from the Southern Appalachians to the current central region, and the other expanded from a Northern Appalachian refugium to form the current eastern and western lineages (Zamudio and Savage 2003). Similarly, expansion from two separate refugia led to the same relationship among regional lineages in the Four‐toed Salamander, *Hemidactylium scutatum* (Herman and Bouzat 2015). An expansion from East to West in *E. lucifuga* is supported not only by the results presented here, but also was inferred by Martin et al. (2015) in an ancestral habitat reconstruction that included a western and an eastern individual.

The chronogram recovered by *BEAST places divergences between major lineages in *E. lucifuga* during the early to mid‐Pleistocene, while divergence among populations within major clades ranges from the late Pleistocene to within the last 10,000 years. Very recent divergence between lineages is also supported by ABC dating estimates. The sharing of haplotypes among localities in concert with the departures from neutrality we observe in estimates of Tajima's *D* and Fu's *F* and reduced nucleotide diversity suggests further recent expansion within the eastern and western regions. Lineage divergence during the turbulent Pleistocene reflects what we know about other North American salamanders (Church et al. 2003; Herman and Bouzat 2015; Newman and Austin 2015) as well as other cave‐dwelling species (Niemiller et al. 2013; Bryson et al. 2014), and in particular, other troglophilic salamanders (Cimmaruta et al. 2015; Kuchta et al. 2016). However, this estimation is much more recent than the dispersal event predicted by Martin et al. (2015), which they estimated to have occurred approximately 4 Ma.

Analysis using microsatellite data tell a slightly different story about genetic structuring across the range of *E. lucifuga*. Major lineages identified using STRUCTURE are similar to those seen in the trees: we see clustering among eastern, western, and central populations. STRUCTURE results also indicate substructuring within the central lineage, between south‐central populations (those located in Tennessee and Kentucky) and north‐central populations (located in Indiana). However, reconstructions of the evolutionary history of these regions using DIYABC indicated that the eastern and central populations diverged most recently, and that the western populations are the most divergent. The timing of these divergence events was predicted to be much more recent in DIYABC than was estimated using gene loci data, which may reflect a tendency for the underestimation of these events when using microsatellite markers, but also may lend support for recent divergence among these lineages.

Population genetic analyses reveal that the majority of genetic variation across the range of *E. lucifuga* is partitioned among the four major geographic regions, and population structure within each region, although significant, is quite low (*R*_ST_ = 0.038). This low structure contrasts with closely related cave‐restricted species (*Eurycea tynerensis*:* F*_ST_ = 0.268, Emel and Bonett 2011; *Eurycea pterophila/E. nana/E. neotenes*:* F*_ST_ = 0.249–0.922, Lucas et al. 2009) and with non‐cave‐dwelling plethodontids studied using protein sequences, in which estimates of *F*_ST_ ranged from 0.13 (*Plethodon cinereus*) to 0.80 (*Plethodon dorsalis dorsalis*) (Larson et al. 1984). However, population structure is similar to estimates seen at smaller scales in *P. cinereus* (*F*_ST_ = 0.019; Cabe et al. 2007) and *A. maculatum* (*F*_ST_ = 0.041; Purrenhage et al. 2009). Low population structure could be a signature of the recent expansion predicted by our demographic statistical analysis. Alternatively, it also suggests the possibility of persistent gene flow among populations. Interestingly, there are distinct differences in population genetic structure and diversity among the main geographic clades of *E. lucifuga*: population structure among populations is only significant within the south‐central and north‐central subregions, while the eastern and western regions exhibit no significant intraregion population structure. This could reflect the inferred recent expansions in the western and eastern regions, which would result in decreased diversity and population structure. Regional differences in dispersal capabilities may also play a part in the variation seen in structure and diversity, as movement between caves is often dependent on the characteristics of the associated surface environment (Caccone 1985; Chiari et al. 2012). Dispersal capabilities in *E. lucifuga* are not well characterized, and among cave‐dwelling species, it has been shown that the extent to which the noncave habitat presents a barrier to dispersal depends greatly on the ecological requirements of the species (Caccone 1985). Some troglophilic cave‐dwellers, such as *H. strinatii* (Chiari et al. 2012; Cimmaruta et al. 2015) and *P. reddelli* (Bryson et al. 2014) show very little gene flow among cave localities. However, other cave‐dwellers have exhibited a great deal of gene flow among caves (Giuseffi et al. 1978; Caccone and Sbordoni 1987), although often this is the result of underground dispersal.

The discordance we see between sequence and microsatellite analyses, which we infer to be a conflict of signal from the mitochondrial and the nuclear DNA due to the minimal signal from the POMC locus, is not uncommon in the literature, and could be indicative of underlying complexities in the evolutionary history of *E. lucifuga*. Most cases where discordance is seen in genetic structure can be attributed to differences in selection or demographic asymmetry among lineages. In the vast majority of such cases (97% of those studied), the taxa in question had experienced secondary contact following divergence (Toews and Belsford, 2012). This is the case in several species of salamanders, including *Chioglossa lusitanica* (Sequeira et al. 2005), *Plethodon glutinosis* and *P. shermani* (Weisrock et al. 2005), *Triturus cristatus* and *T. marmoratus* (Arntzen et al. 2009), *Triturus montandoni* and *T. vulgaris* (Babik et al. 2003), *Salamandra salamandra* (Pereira et al. 2016), *Ambystoma barbouri* and *A. texanum* (Denton et al. 2014), and *Ambystoma macrodactylum* (Lee‐Yaw and Irwin 2012; Lee‐Yaw et al. 2014), where it is hypothesized that after experiencing divergence due to glaciation and climatic shifts, recontact led to introgression one or several times during climatic oscillations of the Pleistocene and more recently. Although discordance between mtDNA and nuclear DNA conflicts in tree building are often resolved in favor of nuclear DNA, Fisher‐Reid and Wiens (2011) observed that among *Plethodon* salamanders, the mitochondrial patterns were favored despite unusually high numbers of strongly supported conflicts. This is similar to the results we observe in *E. lucifuga*, with mitochondrial signal overwhelming the nuclear signal in combined trees.

The results we see in the case of *E. lucifuga* suggest that mitochondrial gene loci reflects a more ancient evolutionary history, wherein the central and eastern/western lineages diverged during the climatic shifts of the Pleistocene, possibly taking refuge in two locations as has been seen in other species (Phillips 1994; Zamudio and Savage 2003; Herman and Bouzat 2015). However, following expansion from Pleistocene refugia and establishment in present‐day localities, there may have been secondary contact between central and eastern populations leading to introgression seen in the microsatellites and the POMC gene trees. Further work is required to explore whether or not gene flow is ongoing between populations; significant population structure in the central regions, but not in the eastern or western region could indicate differing dispersal patterns and therefore asymmetric gene flow among regions, or it could be a signature of recent expansion in the East and West.

## Conflict of Interest

None declared.

## Supporting information


**Figure S1.** Maximum likelihood tree of cyt*b*, on which the major geographic regions are labeled.
**Figure S2.** Maximum likelihood tree of Nd2, on which the major geographic regions are labeled.
**Figure S3.** Maximum likelihood tree of POMC.
**Figure S4.** Divergence points for times estimated using microsatellite data in diyABC.
**Table S1.** Collecting localities and accession numbers.
**Table S2.** Primer sources and sequence for both PCR and sequencing steps, as well as thermocycler conditions for each primer set.
**Table S3.** Summary of the sequencing results and assembly of the two paired‐end libraries.
**Table S4.** Microsatellite markers developed for *Eurycea lucifuga*, reported with primer sequence information as well as multiplex configuration and motif.
**Table S5.** Results of jModelTest indicating the most likely model of substitution for each gene locus.
**Table S6.** Hardy‐Weinberg estimations for each locus within each population.
**Table S7.** Results of Structure Harvester indicating the most likely number of genetic clusters in the microsatellite dataset, highlighted in yellow.
**Table S8.** Mode and quantiles of the posterior distributions of the estimated demographic parameters for the *Eurycea lucifuga* microsatellite dataset using DIYABC.
