# Supplementary material for: Cyto‐nuclear discordance suggests complex evolutionary history in the cave‐dwelling salamander, Eurycea lucifuga
Source: Ecol Evol. 2016 Jul 30;6(17):6121–38. doi: 10.1002/ece3.2212 (PMC5016636; doi:10.1002/ece3.2212)
Supplement: Supplementary file 1 — Figure S1. Maximum likelihood tree of cytb, on which the major geographic regions are labeled. Figure S2. Maximum likelihood tree of Nd2, on which the major geographic regions are labeled. Figure S3. Maximum likelihood tree of POMC. Figure S4. Divergence points for times estimated using microsatellite data in diyABC. Table S1. Collecting localities and accession numbers. Table S2. Primer sources and sequence for both PCR and sequencing steps, as well as thermocycler conditions for each primer set. Table S3. Summary of the sequencing results and assembly of the two paired‐end libraries. Table S4. Microsatellite markers developed for Eurycea lucifuga, reported with primer sequence information as well as multiplex configuration and motif. Table S5. Results of jModelTest indicating the most likely model of substitution for each gene locus. Table S6. Hardy‐Weinberg estimations for each locus within each population. Table S7. Results of Structure Harvester indicating the most likely number of genetic clusters in the microsatellite dataset, highlighted in yellow. Table S8. Mode and quantiles of the posterior distributions of the estimated demographic parameters for the Eurycea lucifuga microsatellite dataset using DIYABC. [file ECE3-6-6121-s001.docx]

**Supplementary Figures**

Fig. S1. Maximum likelihood tree of cytb, on which the major geographic regions are labeled. *Pseudotriton ruber* was included as an outgroup, but is not shown here. Branches are labeled with bootstraps above 50. Bayesian reconstructions supported major regional relationships, and posterior support values above 0.5 from the Bayesian reconstruction are included below branches where appropriate.

Fig. S2. Maximum likelihood tree of Nd2, on which the major geographic regions are labeled. *Pseudotriton ruber* was included as an outgroup, but is not shown here. Branches are labeled with bootstraps above 50. Bayesian reconstructions supported major regional relationships, and posterior support values above 0.5 from the Bayesian reconstruction are included below branches where appropriate.

Fig. S3. Maximum likelihood tree of POMC. *Pseudotriton ruber* was included as an outgroup, but is not shown here. Major regional groups were recovered from POMC data in neither the Maximum Likelihood nor the Bayesian tree. Branches are labeled with bootstraps above 50.

Figure S4. Divergence points for times estimated using microsatellite data in diyABC. Estimates in generation times and years are presented in Table S8.

**Supplementary Tables**

Table S1. Collecting localities and accession numbers

| Sample ID | State | Location | Date | DNA | Microsatellites |
| --- | --- | --- | --- | --- | --- |
| Ad7.1.1 | Kentucky | Adwell | 7/1/13 | no | yes |
| Aus6.30.1 | Kentucky | Austin of Unknown Cave | 6/30/13 | no | yes |
| Aus7.2.1 | Kentucky | Austin | 7/2/13 | no | yes |
| BC7.28.1 | West Virginia | Buckeye Creek | 7/29/12 | no | yes |
| BF7.18.1 | Indiana | Bankley/Fairground | 7/18/12 | yes | yes |
| BF7.18.2 | Indiana | Bankley/Fairground | 7/18/12 | yes | yes |
| BF7.18.3 | Indiana | Bankley/Fairground | 7/18/12 | no | yes |
| BF7.18.4 | Indiana | Bankley/Fairground | 7/18/12 | no | yes |
| BF7.18.5 | Indiana | Bankley/Fairground | 7/18/12 | no | yes |
| BF7.18.6 | Indiana | Bankley/Fairground | 7/18/12 | yes | yes |
| BF7.18.7 | Indiana | Bankley/Fairground | 7/18/12 | yes | yes |
| BF7.18.8 | Indiana | Bankley/Fairground | 7/18/12 | yes | yes |
| BF7.18.9 | Indiana | Bankley/Fairground | 7/18/12 | yes | yes |
| Big7.3.1 | Kentucky | Big Hollow Cave | 7/3/13 | no | yes |
| Big7.3.2 | Kentucky | Big Hollow Cave | 7/3/13 | no | yes |
| Big7.3.3 | Kentucky | Big Hollow Cave | 7/3/13 | no | yes |
| Big7.3.4 | Kentucky | Big Hollow Cave | 7/3/13 | no | yes |
| Big7.3.5 | Kentucky | Big Hollow Cave | 7/3/13 | no | yes |
| Big7.3.6 | Kentucky | Big Hollow Cave | 7/3/13 | no | yes |
| Big7.3.7 | Kentucky | Big Hollow Cave | 7/3/13 | no | yes |
| Bl7.11.1 | Oklahoma | Blue Moon | 7/11/12 | no | yes |
| Bl7.11.5 | Oklahoma | Blue Moon | 7/11/12 | no | yes |
| Bla7.3.1 | Kentucky | Black Rock | 7/3/13 | no | yes |
| BLB9.4.1 | Virginia | Blankenship Blowhole | 9/4/11 | yes | yes |
| BLB9.4.2 | Virginia | Blankenship Blowhole | 9/4/11 | yes | yes |
| BLB9.4.4 | Virginia | Blankenship Blowhole | 9/4/11 | yes | yes |
| BLB9.4.5 | Virginia | Blankenship Blowhole | 9/4/11 | no | yes |
| BLB9.4.6 | Virginia | Blankenship Blowhole | 9/4/11 | no | yes |
| BLB9.4.7 | Virginia | Blankenship Blowhole | 9/4/11 | no | yes |
| Bor6.13.1 | West Virginia | Borehole | 6/13/13 | no | yes |
| Bor6.13.2 | West Virginia | Borehole | 6/13/13 | no | yes |
| Bor7.28.1 | West Virginia | Boarhole | 7/29/12 | no | yes |
| Bor7.28.2 | West Virginia | Boarhole | 7/29/12 | no | yes |
| Bu7.14.1 | Missouri | Bull Creek | 7/14/12 | yes | yes |
| Bu7.14.2 | Missouri | Bull Creek | 7/14/12 | no | yes |
| Bu7.14.3 | Missouri | Bull Creek | 7/14/12 | yes | yes |
| Bu7.14.4 | Missouri | Bull Creek | 7/14/12 | no | yes |
| Bu7.14.5 | Missouri | Bull Creek | 7/14/12 | yes | yes |
| Bu7.14.6 | Missouri | Bull Creek | 7/14/12 | no | yes |
| Bu7.14.7 | Missouri | Bull Creek | 7/14/12 | yes | yes |
| Bu7.14.8 | Missouri | Bull Creek | 7/14/12 | yes | yes |
| Bu7.14.9 | Missouri | Bull Creek | 7/14/12 | yes | yes |
| Byrd10.1.1 | Virginia | Byrd's water | 10/1/11 | no | yes |
| Byrd10.1.11 | Virginia | Byrd's water | 10/1/11 | yes | yes |
| Byrd10.1.13 | Virginia | Byrd's water | 10/1/11 | yes | yes |
| Byrd10.1.14 | Virginia | Byrd's water | 10/1/11 | yes | yes |
| Byrd10.1.15 | Virginia | Byrd's water | 10/1/11 | no | yes |
| Byrd10.1.16 | Virginia | Byrd's water | 10/1/11 | yes | yes |
| Byrd10.1.3 | Virginia | Byrd's water | 10/1/11 | yes | yes |
| Byrd10.1.4 | Virginia | Byrd's water | 10/1/11 | no | yes |
| Byrd10.1.5 | Virginia | Byrd's water | 10/1/11 | no | yes |
| Byrd10.1.6 | Virginia | Byrd's water | 10/1/11 | no | yes |
| C27.1.1 | Kentucky | Frank's Cave | 7/1/13 | no | yes |
| C27.1.2 | Kentucky | Frank's Cave | 7/1/13 | no | yes |
| C27.1.3 | Kentucky | Frank's Cave | 7/1/13 | no | yes |
| C27.1.4 | Kentucky | Frank's Cave | 7/1/13 | no | yes |
| C27.2.1 | Kentucky | Frank's Cave | 7/2/13 | no | yes |
| C27.2.2 | Kentucky | Frank's Cave | 7/2/13 | no | yes |
| C27.2.3 | Kentucky | Frank's Cave | 7/2/13 | no | yes |
| Cad6.30.1 | Kentucky | Cadaverous | 6/30/13 | no | yes |
| Cad6.30.2 | Kentucky | Cadaverous | 6/30/13 | no | yes |
| Cad6.30.3 | Kentucky | Cadaverous | 6/30/13 | no | yes |
| Cad6.30.4 | Kentucky | Cadaverous | 6/30/13 | no | yes |
| Cad6.30.5 | Kentucky | Cadaverous | 6/30/13 | no | yes |
| Cr6.7.2 | Tennessee | Crews | 6/7/12 | no | yes |
| Cr6.7.3 | Tennessee | Crews | 6/7/12 | no | yes |
| Cr6.7.4 | Tennessee | Crews | 6/7/12 | no | yes |
| Cr6.7.5 | Tennessee | Crews | 6/7/12 | no | yes |
| Cr7.13.1 | Missouri | Crighton Spring | 7/13/12 | yes | yes |
| Cr7.13.2 | Missouri | Crighton Spring | 7/13/12 | no | yes |
| Cr7.13.3 | Missouri | Crighton Spring | 7/13/12 | no | yes |
| Cr7.13.4 | Missouri | Crighton Spring | 7/13/12 | yes | yes |
| Cr7.13.5 | Missouri | Crighton Spring | 7/13/12 | yes | yes |
| Crys6.30.1 | Kentucky | Crystal Cave | 6/30/13 | no | yes |
| Crys6.30.2 | Kentucky | Crystal Cave | 6/30/13 | no | yes |
| Cu6.13.2 | West Virginia | Balfour to Culver's Cave | 6/13/13 | no | yes |
| D7.19.1 | Indiana | Donnehue | 7/19/12 | yes | yes |
| D7.19.2 | Indiana | Donnehue | 7/19/12 | yes | yes |
| D7.19.3 | Indiana | Donnehue | 7/19/12 | yes | yes |
| D7.19.4 | Indiana | Donnehue | 7/19/12 | yes | yes |
| D7.19.5 | Indiana | Donnehue | 7/19/12 | yes | yes |
| E7.20.1 | Indiana | Lost Cave | 7/20/12 | no | yes |
| E7.20.2 | Indiana | Lost Cave | 7/20/12 | yes | yes |
| E7.20.3 | Indiana | Lost Cave | 7/20/12 | yes | yes |
| E7.20.4 | Indiana | Lost Cave | 7/20/12 | yes | yes |
| E7.20.5 | Indiana | Lost Cave | 7/20/12 | yes | yes |
| E7.20.6 | Indiana | Lost Cave | 7/20/12 | yes | yes |
| Fall6.30.1 | Kentucky | Falling Tree | 6/30/13 | no | yes |
| Fall6.30.2 | Kentucky | Falling Tree | 6/30/13 | no | yes |
| Fall6.30.3 | Kentucky | Falling Tree | 6/30/13 | no | yes |
| Fall7.3.1 | Kentucky | Falling Tree | 7/3/13 | no | yes |
| Fall7.5.1 | Kentucky | Falling Tree | 7/5/13 | no | yes |
| Fall7.5.2 | Kentucky | Falling Tree | 7/5/13 | no | yes |
| G6.5.1 | Tennessee | Gillespie | 6/5/12 | no | yes |
| G6.5.2 | Tennessee | Gillespie | 6/5/12 | no | yes |
| G6.5.3 | Tennessee | Gillespie | 6/5/12 | no | yes |
| G6.5.4 | Tennessee | Gillespie | 6/5/12 | no | yes |
| GrOn6.29.1 | Kentucky | Great Onyx | 6/29/13 | no | yes |
| GrOn6.29.10 | Kentucky | Great Onyx | 6/29/13 | no | yes |
| GrOn6.29.11 | Kentucky | Great Onyx | 6/29/13 | no | yes |
| GrOn6.29.2 | Kentucky | Great Onyx | 6/29/13 | no | yes |
| GrOn6.29.3 | Kentucky | Great Onyx | 6/29/13 | no | yes |
| GrOn6.29.4 | Kentucky | Great Onyx | 6/29/13 | no | yes |
| GrOn6.29.5 | Kentucky | Great Onyx | 6/29/13 | no | yes |
| GrOn6.29.6 | Kentucky | Great Onyx | 6/29/13 | no | yes |
| GrOn6.29.7 | Kentucky | Great Onyx | 6/29/13 | no | yes |
| GrOn6.29.8 | Kentucky | Great Onyx | 6/29/13 | no | yes |
| GrOn6.29.9 | Kentucky | Great Onyx | 6/29/13 | no | yes |
| H6.13.1 | West Virginia | Higganbotham | 6/13/13 | no | yes |
| H6.13.2 | West Virginia | Higganbotham | 6/13/13 | no | yes |
| H7.28.1 | West Virginia | Higginbothom #1 | 7/29/12 | yes | yes |
| H7.28.2 | West Virginia | Higginbothom #2 | 7/29/12 | yes | yes |
| Hick7.3.1 | Kentucky | Hickory Cabin | 7/3/13 | no | yes |
| Hick7.3.2 | Kentucky | Hickory Cabin | 7/3/13 | no | yes |
| Hick7.3.3 | Kentucky | Hickory Cabin | 7/3/13 | no | yes |
| Hick7.3.4 | Kentucky | Hickory Cabin | 7/3/13 | no | yes |
| Hick7.3.5 | Kentucky | Hickory Cabin | 7/3/13 | no | yes |
| Hick7.3.6 | Kentucky | Hickory Cabin | 7/3/13 | no | yes |
| His6.29.1 | Kentucky | Historic entrance | 6/29/13 | no | yes |
| His6.29.2 | Kentucky | Historic entrance | 6/29/13 | no | yes |
| IG7.10.1 | Oklahoma | Iron Gate | 7/10/12 | no | yes |
| IG7.10.2 | Oklahoma | Iron Gate | 7/10/12 | no | yes |
| IG7.10.3 | Oklahoma | Iron Gate | 7/10/12 | no | yes |
| J7.10.1 | Oklahoma | Jail | 7/10/12 | yes | yes |
| J7.10.2 | Oklahoma | Jail | 7/10/12 | no | yes |
| J7.10.3 | Oklahoma | Jail | 7/10/12 | no | yes |
| J7.10.4 | Oklahoma | Jail | 7/10/12 | yes | yes |
| J7.10.5 | Oklahoma | Jail | 7/10/12 | yes | yes |
| J7.10.6 | Oklahoma | Jail | 7/10/12 | yes | yes |
| J7.10.7 | Oklahoma | Jail | 7/10/12 | no | yes |
| J7.10.8 | Oklahoma | Jail | 7/10/12 | no | yes |
| J7.10.9 | Oklahoma | Jail | 7/10/12 | no | yes |
| Jan7.9.1 | Oklahoma | January-Stansbury | 7/9/12 | yes | yes |
| Jan7.9.2 | Oklahoma | January-Stansbury | 7/9/12 | yes | yes |
| Jan7.9.3 | Oklahoma | January-Stansbury | 7/9/12 | no | yes |
| Jan7.9.4 | Oklahoma | January-Stansbury | 7/9/12 | no | yes |
| Jan7.9.5 | Oklahoma | January-Stansbury | 7/9/12 | yes | yes |
| Jan7.9.6 | Oklahoma | January-Stansbury | 7/9/12 | yes | yes |
| Jan7.9.7 | Oklahoma | January-Stansbury | 7/9/12 | no | yes |
| Left7.3.1 | Kentucky | Left eye | 7/3/13 | no | yes |
| Lil7.2.1 | Kentucky | Little Beauty | 7/2/13 | no | yes |
| Lil7.2.2 | Kentucky | Little Beauty | 7/2/13 | no | yes |
| LP6.3.4 | Tennessee | Lost Puddle | 6/3/12 | no | yes |
| Man7.28.1 | West Virginia | Mann | 7/29/12 | no | yes |
| Man7.28.2 | West Virginia | Mann | 7/29/12 | yes | yes |
| Man7.28.3 | West Virginia | Mann | 7/29/12 | yes | yes |
| Nat7.3.1 | Kentucky | Natural Bridge | 7/3/13 | no | yes |
| New6.29.1 | Kentucky | New Discovery | 6/29/13 | no | yes |
| New6.29.2 | Kentucky | New Discovery | 6/29/13 | no | yes |
| New6.29.3 | Kentucky | New Discovery | 6/29/13 | no | yes |
| New6.29.4 | Kentucky | New Discovery | 6/29/13 | no | yes |
| New6.29.5 | Kentucky | New Discovery | 6/29/13 | no | yes |
| New7.5.1 | Kentucky | New Discovery | 7/5/13 | no | yes |
| New7.5.2 | Kentucky | New Discovery | 7/5/13 | no | yes |
| New7.5.3 | Kentucky | New Discovery | 7/5/13 | no | yes |
| New7.5.4 | Kentucky | New Discovery | 7/5/13 | no | yes |
| New7.5.5 | Kentucky | New Discovery | 7/5/13 | no | yes |
| New7.5.6 | Kentucky | New Discovery | 7/5/13 | no | yes |
| Oe6.7.1 | Tennessee | Eoff | 6/7/12 | no | yes |
| Oe6.7.2 | Tennessee | Eoff | 6/7/12 | no | yes |
| Oe6.7.4 | Tennessee | Eoff | 6/7/12 | no | yes |
| Oe6.7.6 | Tennessee | Eoff | 6/7/12 | no | yes |
| Oe6.7.7 | Tennessee | Eoff | 6/7/12 | no | yes |
| Oe6.7.8 | Tennessee | Eoff | 6/7/12 | no | yes |
| Pag7.6.11 | Kentucky | Pagoda | 7/6/13 | no | yes |
| Pag7.6.2 | Kentucky | Pagoda | 7/6/13 | no | yes |
| Pag7.6.3 | Kentucky | Pagoda | 7/6/13 | no | yes |
| Phil7.3.1 | Kentucky | Phil | 7/3/13 | no | yes |
| Phil7.3.2 | Kentucky | Phil | 7/3/13 | no | yes |
| Phil7.3.3 | Kentucky | Phil | 7/3/13 | no | yes |
| Phil7.3.4 | Kentucky | Phil | 7/3/13 | no | yes |
| Pom6.6.2 | Tennessee | Pompie | 6/6/12 | yes | yes |
| Pom6.6.3 | Tennessee | Pompie | 6/6/12 | yes | yes |
| Pr.6.6.3 | Tennessee | Prowell | 6/6/12 | no | yes |
| Pr6.6.1 | Tennessee | Prowell | 6/6/12 | yes | yes |
| Pr6.6.2 | Tennessee | Prowell | 6/6/12 | yes | yes |
| Pr6.6.4 | Tennessee | Prowell | 6/6/12 | no | yes |
| Pr6.6.5 | Tennessee | Prowell | 6/6/12 | yes | yes |
| Pr6.6.6 | Tennessee | Prowell | 6/6/12 | yes | yes |
| RC7.19.1 | Indiana | Roberts | 7/19/12 | yes | yes |
| RC7.19.2 | Indiana | Roberts | 7/19/12 | yes | yes |
| RC7.19.3 | Indiana | Roberts | 7/19/12 | yes | yes |
| RC7.19.4 | Indiana | Roberts | 7/19/12 | yes | yes |
| RL7.18.1 | Indiana | Robinson Ladder | 7/18/12 | yes | yes |
| RL7.18.2 | Indiana | Robinson Ladder | 7/18/12 | yes | yes |
| RL7.18.3 | Indiana | Robinson Ladder | 7/18/12 | yes | yes |
| RL7.18.4 | Indiana | Robinson Ladder | 7/18/12 | yes | yes |
| S7.11.1 | Oklahoma | Survivalist* | 7/11/12 | no | yes |
| S7.11.2 | Oklahoma | Survivalist* | 7/11/12 | no | yes |
| Sil7.6.1 | Kentucky | Silent Spring | 7/6/13 | no | yes |
| Sil7.6.2 | Kentucky | Silent Spring | 7/6/13 | no | yes |
| Sil7.6.3 | Kentucky | Silent Spring | 7/6/13 | no | yes |
| Sil7.6.4 | Kentucky | Silent Spring | 7/6/13 | no | yes |
| Sil7.6.5 | Kentucky | Silent Spring | 7/6/13 | no | yes |
| Sil7.6.6 | Kentucky | Silent Spring | 7/6/13 | no | yes |
| Smoke1 | Virginia | Smokehole |  | yes | yes |
| Smoke2 | Virginia | Smokehole |  | yes | yes |
| Smoke9.3.1 | Virginia | Smokehole | 9/3/11 | no | yes |
| Smoke9.3.2 | Virginia | Smokehole | 9/3/11 | yes | yes |
| Smoke9.3.3 | Virginia | Smokehole | 9/3/11 | no | yes |
| Smoke9.3.4 | Virginia | Smokehole | 9/3/11 | yes | yes |
| Spr7.28.1 | West Virginia | Spring | 7/29/12 | no | yes |
| Stan7.2.1 | Kentucky | Stan's Well | 7/2/13 | no | yes |
| Stan7.3.1 | Kentucky | Stan's Well | 7/3/13 | no | yes |
| Stur7.3.1 | Kentucky | Sturgeon | 7/3/13 | no | yes |
| Su7.20.1 | Indiana | Sullivan | 7/20/12 | yes | yes |
| Su7.20.2 | Indiana | Sullivan | 7/20/12 | yes | yes |
| Su7.20.3 | Indiana | Sullivan | 7/20/12 | yes | yes |
| Su7.20.4 | Indiana | Sullivan | 7/20/12 | no | yes |
| Su7.20.5 | Indiana | Sullivan | 7/20/12 | yes | yes |
| Su7.20.6 | Indiana | Sullivan | 7/20/12 | no | yes |
| Taw9.24.1 | Virginia | Tawney's | 9/24/11 | yes | yes |
| Taw9.24.2 | Virginia | Tawney's | 9/24/11 | yes | yes |
| Taw9.24.3 | Virginia | Tawney's | 9/24/11 | yes | yes |
| Th7.11.1 | Oklahoma | Third | 7/11/12 | no | yes |
| Th7.11.2 | Oklahoma | Third | 7/11/12 | yes | yes |
| Th7.11.3 | Oklahoma | Third | 7/11/12 | yes | yes |
| Th7.11.4 | Oklahoma | Third | 7/11/12 | no | yes |
| Vici6.29.1 | Kentucky | Violet City | 6/29/13 | no | yes |
| Vici6.29.2 | Kentucky | Violet City | 6/29/13 | no | yes |
| Vici6.29.3 | Kentucky | Violet City | 6/29/13 | no | yes |
| Vici6.29.4 | Kentucky | Violet City | 6/29/13 | no | yes |
| Vici6.29.5 | Kentucky | Violet City | 6/29/13 | no | yes |
| Vici6.29.6 | Kentucky | Violet City | 6/29/13 | no | yes |
| Whi7.1.1 | Kentucky | White | 7/1/13 | no | yes |
| Whi7.1.2 | Kentucky | White | 7/1/13 | no | yes |
| YMCA7.4.1 | Kentucky | YMCA | 7/4/13 | no | yes |
| YMCA7.4.2 | Kentucky | YMCA | 7/4/13 | no | yes |
| YMCA7.4.3 | Kentucky | YMCA | 7/4/13 | no | yes |
| YMCA7.4.4 | Kentucky | YMCA | 7/4/13 | no | yes |
| YMCA7.4.5 | Kentucky | YMCA | 7/4/13 | no | yes |
| YMCA7.4.6 | Kentucky | YMCA | 7/4/13 | no | yes |
| YMCA7.4.7 | Kentucky | YMCA | 7/4/13 | no | yes |
| YMCA7.4.8 | Kentucky | YMCA | 7/4/13 | no | yes |

Table S2. Primer sources and sequence for both PCR and sequencing steps, as well as thermocycler conditions for each primer set.

| **Primer** | **Source** | **Sequence** | **Cycling conditions** |
| --- | --- | --- | --- |
| cyt*b*F | Harlan and Zigler (2009) | AAGATTATTAATAACTCCTTTATTGA | Annealing temp. of 50C, 35 cycles |
| cyt*b*R |  | AAAATGCTTGTCCAATTTCAAT |  |
| ND2F | This study | TACAAGCCTCAGCATCTGCC | Annealing temp. of 59.4C, 30 cycles |
| ND2R |  | ATCCAGAGGTTGGTGGGAGT |  |
| POMCF | Lamb *et al.* (2012) | ATATGTCATGAGCCATTTTCGCTGGAA | Annealing temp. of 58C, 45 cycles |
| POMCR |  | GGCATTTTTGAAAAGAGTCATTAGAGG |  |

Table S3. Summary of the sequencing results and assembly of the two paired-end libraries.

| **Library** | **Number of reads** | **Q>30** | **Total transcripts** | **Median contig length** | **Total assembled bases** |
| --- | --- | --- | --- | --- | --- |
| BLB | 128 mill | 89.96% | 112,449 | 355bp | 73,398,489 |
| Smoke | 300 mill | 90.26% | 92,594 | 362bp | 59,509,037 |

Table S4. Microsatellite markers developed for *Eurycea lucifuga*, reported with primer sequence information as well as multiplex configuration and motif. We characterized the number of alleles at each locus (*k*), as well as observed (H_o_) and expected (H_s_) heterozygosity.

| **Marker name** | **Forward primer** | **Reverse Primer** | **Plex** | **Motif** | ***k*** | **Ho** | **Hs** |
| --- | --- | --- | --- | --- | --- | --- | --- |
| E_luc_238 | ATGGCTGCGCTTTCTTGTAC | CTCTGTACAGGAGACGGGTG | 1 | AAG | 3 | 0.009 | 0.009 |
| E_luc_915 | TGCCGAAAGTTGCAGTGAAG | CGCATCGTCATCTGCAGAAG | 1 | ATC | 4 | 0.04 | 0.048 |
| E_luc_1405 | ACTGAGCAAACTTCGCATGG | TGTCCAGATGCCTCTACAGC | 1 | ACCT | 5 | 0.092 | 0.099 |
| E_luc_1259 | ACAGCTTGCTTACTTGGTGC | AAGGGAACAAGGCTCAGAGG | 2 | AAG | 4 | 0.473 | 0.519 |
| E_luc_1375 | ACAAGCTCCATTTGCACGAG | GTGGTAGCCCTGGTTCTAGG | 2 | AACC | 5 | 0.13 | 0.175 |
| E_luc_1284 | GGTCTTTGTCAGCAGTGCAG | CCGAGGGCCTAAGTCTAACC | 2 | AAGC | 5 | 0.194 | 0.273 |
| E_luc_423 | GGATGAAGAAGGGTACTGCG | GCTGACTCTTGCAGACTGTG | 3 | ACC | 3 | 0.198 | 0.204 |
| E_luc_1055 | TGTGGTTGTATGCTTATCAGGC | TTCTGTGTGCTCAAGGAGATG | 3 | AAT | 3 | 0.094 | 0.205 |
| E_luc_433 | TGGAAAGGAAGCCAAAGTCAC | GTGCCAAATCCCTCTGCATC | 4 | AAT | 4 | 0.107 | 0.267 |
| E_luc_971 | CAGCCACAATCCAAGAACCC | AAGCCGGAATAGTAGAGCCG | 4 | ATC | 7 | 0.431 | 0.543 |
| E_luc_240 | TGCTATGACCTCTGGCATCC | AAGTTCTCCAGAGGCCTTGG | 4 | AAAT | 2 | 0.174 | 0.25 |
| E_luc_2440 | GCAGCAGAAACAAGGACTGG | CCAGTCTGACAGTGCGGG | 4 | AGGG | 7 | 0.211 | 0.286 |
| E_luc_371 | GTATGTGTGCACTGCGAGAG | TCAGTGGCTTGGATCTGGTG | 5 | ACC | 5 | 0.033 | 0.04 |
| E_luc_2319 | ATCAACGTTCTGAATGCGCC | TGCACTGAACTAGGAGGGAC | 5 | AAT | 5 | 0.361 | 0.381 |
| E_luc_961 | TGTTGCAAAGTTCTGGTCGG | CGTGCTTTACTTCCTTGGCC | 5 | ACC | 4 | 0.206 | 0.215 |
| E_luc_808 | CCCAGAACATGCACAACCAG | TAGCGGCTGGAAGAAGGATC | 6 | AGC | 5 | 0.655 | 0.499 |
| E_luc_2336 | TTTCATGGCTGCTTGTACCC | ACATACTACAACTCGAGGTGC | 6 | AAAT | 3 | 0.04 | 0.046 |
| E_luc_566 | AGGGTTTAACTGCTGAAGGG | GCAAATCTCAGCCGTGTCTC | 6 | AAAT | 4 | 0.04 | 0.198 |
| E_luc_2121 | CCCTCCCTGTGCTTACTCTG | ACGATCTGACCTGATGACCG | 6 | AATT | 2 | 0.014 | 0.013 |

| Table S5. Results of jModelTest indicating the most likely model of substitution for each gene locus.   \| **Locus** \| **AIC** \| **BIC** \| **DT** \| \| --- \| --- \| --- \| --- \| \| *cytb* \| TVM+IG \| TPMuf+IG \| TPMuf+IG \| \| ND2 \| GTR+I \| TIM3+I \| TIM3+I \| \| POMC \| TIM2+G \| TrN \| TrN \|   Table S6. Hardy-Weinberg estimations for each locus within each population. Significant departures from Hardy-Weinberg are indicated in bold. | | | | | | | | | | | | | | | | | | | | |
| --- | --- | --- | --- | --- | --- | --- | --- | --- | --- | --- | --- | --- | --- | --- | --- | --- | --- | --- | --- | --- | --- | --- | --- | --- | --- | --- | --- | --- | --- | --- | --- | --- | --- | --- | --- | --- |
| **Population** | **238** | **915** | **1405** | **1259** | **1375** | **1284** | **423** | **1055** | **433** | **971** | **240** | **2440** | **371** | **2319** | **961** | **808** | **2336** | **566** | **2121** | **Multi-locus** |
| **Adwell** | 0 | 0 | 0 | 0 | 0 | 0 | 0 | 0 | 0 | 0 | 0 | 0 | 0 | 0 | 0 | 0 | 0 | 0 | 0 | --- |
| **Austen** | --- | 0 | 0 | 0 | 0 | 0 | --- | 1 | 0.5 | 0 | 0 | 0 | --- | 0 | 0 | 0 | --- | --- | --- | 0.25 |
| **Buckeye_Creek** | 0 | 0 | 0 | 0 | 0 | 0 | 0 | 0 | 0 | 0 | 0 | 0 | 0 | 0 | 0 | 0 | 0 | 0 | 0 | --- |
| **Bankley** | --- | --- | -0.091 | 0.216 | --- | -0.231 | -0.212 | 0 | --- | -0.116 | 0.158 | -0.067 | --- | 0 | -0.164 | 0.048 | --- | **0.536** | --- | 0.034 |
| **Big** | --- | --- | 0 | 0.321 | 0 | -0.125 | 0 | 1 | 0.647 | 0.419 | 0.368 | -0.358 | --- | 0.213 | -0.125 | -0.2 | --- | 1 | --- | 0.214 |
| **Blue_Moon** | --- | --- | 0 | --- | 0 | --- | --- | --- | --- | -1 | --- | 0 | --- | --- | --- | -1 | --- | --- | --- | -0.5 |
| **Black** | 0 | 0 | 0 | 0 | 0 | 0 | 0 | 0 | 0 | 0 | 0 | 0 | 0 | 0 | 0 | 0 | 0 | 0 | 0 | --- |
| **Blankenship** | --- | --- | --- | 0.062 | --- | --- | --- | --- | 0.5 | 0.524 | --- | --- | --- | --- | --- | -0.538 | 0.333 | --- | --- | 0.141 |
| **Borehole** | --- | --- | --- | 0 | --- | --- | --- | --- | --- | 0.5 | --- | --- | --- | --- | --- | -0.5 | --- | 1 | 0 | 0.25 |
| **Bull_Creek** | --- | --- | --- | -0.333 | -0.44 | --- | --- | --- | **0.59** | -0.514 | --- | --- | 0 | -0.067 | --- | -0.778 | --- | 0.636 | --- | -0.173 |
| **Byrd** | --- | 0.64 | 1 | 0.237 | 0.64 | 1 | --- | 1 | **0.673** | 0.308 | 0 | 0.64 | 0.654 | 0.64 | 0.5 | -0.191 | 0.386 | 1 | 0 | 0.463 |
| **C2** | --- | --- | 0 | -0.429 | 1 | -0.111 | 0 | -0.091 | 0.5 | 0 | 0.727 | 0.41 | --- | 0.368 | 0.122 | 0.294 | --- | --- | --- | 0.252 |
| **Cadaverous** | --- | --- | --- | -0.5 | 1 | 0.636 | -0.143 | 1 | 0.5 | -0.333 | -0.6 | 1 | --- | --- | -0.2 | -0.067 | --- | --- | --- | 0.197 |
| **Crews** | --- | --- | --- | --- | 0 | -1 | 0 | --- | --- | -1 | --- | --- | 0 | 0 | --- | 0 | --- | --- | --- | -0.286 |
| **Crystal** | --- | --- | --- | 1 | --- | 0 | 0 | --- | 0 | --- | 0 | --- | --- | -1 | 0 | --- | --- | --- | --- | 0.125 |
| **Crighton** | --- | --- | 0 | -0.143 | 1 | --- | --- | --- | 1 | 0.273 | --- | 0.6 | --- | --- | --- | -1 | 0 | --- | --- | 0.226 |
| **Culver** | 0 | 0 | 0 | 0 | 0 | 0 | 0 | 0 | 0 | 0 | 0 | 0 | 0 | 0 | 0 | 0 | 0 | 0 | 0 | --- |
| **Donnehue** | --- | --- | --- | -0.333 | --- | 0.6 | -0.143 | 0 | 1 | **0.75** | 0.6 | --- | --- | --- | --- | 0.04 | --- | 1 | --- | 0.432 |
| **Lost** | --- | --- | --- | -0.282 | 0 | 0.487 | -0.154 | --- | --- | -0.25 | -0.25 | --- | --- | --- | -0.111 | 0.211 | --- | 0.643 | --- | 0.046 |
| **Fall** | --- | --- | 0 | 0.318 | 0 | -0.053 | --- | 0.615 | 0 | 0.211 | 0.615 | 0.211 | --- | 0.024 | -0.111 | 0.118 | --- | --- | --- | 0.204 |
| **Gillespie** | --- | 0 | -0.091 | --- | -0.091 | --- | 1 | --- | --- | 0.182 | --- | 0.571 | 0 | 0 | 0 | 0.143 | --- | --- | --- | 0.2 |
| **Great_Onyx** | --- | --- | 0 | -0.033 | 0 | 0.304 | 0.268 | 0.574 | 0.223 | 0.459 | 0.259 | 0.231 | --- | 0.32 | -0.026 | -0.19 | 0 | **1** | --- | 0.237 |
| **Higganbotham** | --- | --- | --- | -1 | 0 | --- | --- | --- | --- | 0.6 | --- | 0 | --- | --- | --- | 0 | --- | --- | --- | 0 |
| **Higganbotham2** | 0 | 0 | 0 | 0 | 0 | 0 | 0 | 0 | 0 | 0 | 0 | 0 | 0 | 0 | 0 | 0 | 0 | 0 | 0 | --- |
| **Hickory** | --- | --- | 0 | -0.163 | --- | -0.143 | 1 | -0.111 | --- | 1 | -0.25 | -0.053 | --- | 0.259 | 0 | 0.25 | --- | --- | --- | 0.128 |
| **Historical** | --- | --- | 0 | 0 | 0 | 0 | 0 | --- | 0 | 0.5 | 1 | -1 | --- | -0.333 | 0.5 | -0.333 | --- | --- | --- | 0.154 |
| **Iron_Gate** | --- | --- | --- | --- | 0 | --- | --- | --- | --- | 0 | --- | 1 | --- | --- | 0 | -1 | --- | 1 | --- | 0.333 |
| **Jail** | --- | -0.067 | --- | -0.032 | -0.2 | --- | --- | --- | **1** | -0.366 | --- | 0 | --- | 0 | 1 | **-0.8** | --- | **0.644** | --- | 0.028 |
| **January** | --- | -0.091 | --- | 0 | 1 | --- | --- | --- | 0.5 | -0.714 | --- | --- | --- | -0.2 | --- | **-1** | --- | 1 | --- | 0.016 |
| **Left** | 0 | 0 | 0 | 0 | 0 | 0 | 0 | 0 | 0 | 0 | 0 | 0 | 0 | 0 | 0 | 0 | 0 | 0 | 0 | --- |
| **Little** | --- | --- | --- | 0 | --- | 0 | 0 | --- | 0.5 | 1 | 1 | 0 | --- | 0.5 | --- | 0 | --- | --- | --- | 0.545 |
| **Puddle** | 0 | 0 | 0 | 0 | 0 | 0 | 0 | 0 | 0 | 0 | 0 | 0 | 0 | 0 | 0 | 0 | 0 | 0 | 0 | --- |
| **Mann** | --- | --- | --- | 1 | --- | --- | 0 | --- | --- | 0.2 | --- | --- | --- | --- | --- | -1 | --- | 0.6 | --- | 0.263 |
| **Natural** | 0 | 0 | 0 | 0 | 0 | 0 | 0 | 0 | 0 | 0 | 0 | 0 | 0 | 0 | 0 | 0 | 0 | 0 | 0 | --- |
| **New** | --- | --- | 0 | 0.151 | --- | 0.64 | 0 | 0.31 | 0.5 | 0.474 | 0.259 | -0.163 | --- | 0.124 | 0.268 | -0.149 | --- | --- | --- | 0.209 |
| **Eoff** | --- | 0 | --- | 0.706 | -0.053 | -0.053 | -0.111 | -0.429 | --- | 0.091 | --- | 0 | 0 | 0.362 | 0.062 | -0.395 | --- | 0 | --- | 0.043 |
| **Pagoda** | --- | 0 | --- | -0.333 | --- | 0 | --- | 1 | 1 | --- | 0 | --- | --- | -0.333 | -0.143 | 0 | --- | --- | --- | 0.216 |
| **Phil** | --- | --- | 0 | 0.294 | --- | 0 | -0.2 | 1 | 1 | 0 | 0 | -0.091 | --- | -0.412 | 0 | 0 | --- | --- | --- | 0.165 |
| **Pompie** | --- | --- | 0 | 1 | --- | 0 | 0 | --- | --- | 0.5 | --- | 0 | 0 | -1 | --- | 0 | --- | 1 | --- | 0.308 |
| **Prowell** | -0.154 | --- | -0.111 | -0.667 | -0.25 | 0.615 | -0.429 | 0.394 | 0.286 | 0.286 | --- | -0.081 | --- | -0.19 | --- | -0.25 | --- | 1 | 0 | 0.024 |
| **Roberts** | --- | --- | --- | 0.571 | --- | 1 | -0.2 | 1 | --- | 0.667 | 0 | --- | --- | --- | --- | -0.5 | --- | 0 | --- | 0.362 |
| **Robinson** | --- | --- | --- | -0.059 | --- | 0.294 | -0.2 | --- | 1 | 0.625 | 0 | --- | --- | --- | -0.2 | -0.412 | --- | --- | --- | 0.174 |
| **Survivalist** | --- | --- | --- | -1 | 0 | --- | --- | --- | --- | -1 | --- | 1 | --- | 0 | --- | -1 | --- | --- | --- | -0.167 |
| **Silver** | --- | --- | --- | -0.391 | 0.615 | 1 | -0.25 | 0.333 | 0.5 | 0 | 0.615 | -0.053 | --- | 0.302 | -0.154 | 0 | --- | --- | --- | 0.21 |
| **Smoke** | --- | --- | --- | 0.706 | --- | --- | 0 | --- | --- | 0.211 | --- | --- | --- | --- | --- | -0.25 | --- | --- | --- | 0.18 |
| **Spring** | 0 | 0 | 0 | 0 | 0 | 0 | 0 | 0 | 0 | 0 | 0 | 0 | 0 | 0 | 0 | 0 | 0 | 0 | 0 | --- |
| **Stan** | --- | --- | 0 | -1 | --- | 1 | --- | 0 | --- | --- | 0 | 0 | --- | -0.333 | --- | 0 | --- | --- | --- | 0.053 |
| **Sturgeon** | 0 | 0 | 0 | 0 | 0 | 0 | 0 | 0 | 0 | 0 | 0 | 0 | 0 | 0 | 0 | 0 | 0 | 0 | 0 | --- |
| **Sullivan** | --- | --- | --- | -0.081 | --- | -0.429 | -0.111 | --- | 1 | 0.211 | 0.062 | --- | --- | --- | -0.111 | 0 | --- | **0.706** | --- | 0.149 |
| **Tawney** | --- | --- | --- | 1 | --- | --- | 0 | --- | --- | 0.2 | --- | --- | --- | --- | --- | 0.111 | 0 | --- | --- | 0.314 |
| **Third** | --- | --- | --- | --- | 0 | --- | --- | --- | --- | -0.2 | --- | --- | --- | -0.2 | --- | -1 | --- | 1 | --- | -0.071 |
| **Violet** | --- | --- | --- | 0.524 | 0 | -0.111 | --- | 0.333 | 0.5 | --- | -0.25 | 0.062 | 0 | -0.081 | -0.111 | --- | --- | 0.062 | --- | 0.121 |
| **White** | --- | --- | --- | 0 | --- | 0 | --- | --- | --- | 0 | -1 | 0.5 | --- | 1 | 0 | --- | --- | --- | --- | 0.222 |
| **YMCA** | --- | --- | --- | -0.111 | --- | -0.077 | -0.167 | -0.077 | 0.65 | 0.192 | 0.741 | -0.273 | --- | 0.079 | -0.077 | -0.105 | --- | --- | --- | 0.089 |
| **Overall** | 0.011 | 0.165 | 0.077 | 0.087 | 0.257 | 0.29 | 0.045 | 0.541 | 0.597 | 0.197 | 0.303 | 0.261 | 0.164 | 0.051 | 0.042 | -0.31 | 0.094 | 0.8 | 0.114 | 0.18 |

Table S7. Results of Structure Harvester indicating the most likely number of genetic clusters in the microsatellite dataset, highlighted in yellow. Results are shown for both partitions in the hierarchical analysis, indicating that the best supported number of clusters (K) is 5.

| **Partition 1** | | | | | | |
| --- | --- | --- | --- | --- | --- | --- |
| K | Reps | Mean LnP(K) | Stdev LnP(K) | Ln'(K) | \|Ln''(K)\| | Delta K |
| 1 | 2 | -4013.4 | 0 | NA | NA | NA |
| 2 | 2 | -3629.95 | 0.6364 | 383.45 | 170.55 | 267.99347 |
| 3 | 2 | -3417.05 | 0.2121 | 212.9 | 86.65 | 408.472017 |
| 4 | 2 | -3290.8 | 1.4142 | 126.25 | 50.75 | 35.885669 |
| 5 | 2 | -3215.3 | 1.5556 | 75.5 | 5.45 | 3.503393 |
| 6 | 2 | -3145.25 | 0.7778 | 70.05 | 51.3 | 65.953778 |
| 7 | 2 | -3126.5 | 0.2828 | 18.75 | 111.9 | 395.626244 |
| 8 | 2 | -3219.65 | 29.2035 | -93.15 | 118.25 | 4.049171 |
| 9 | 2 | -3194.55 | 50.841 | 25.1 | 62.6 | 1.23129 |
| 10 | 2 | -3232.05 | 64.276 | -37.5 | NA | NA |
| **Partition 2** | | | | | | |
| 1 | 2 | -460.2 | 0 | NA | NA | NA |
| 2 | 2 | -494.7 | 1.1314 | -34.5 | 25.65 | 22.671611 |
| 3 | 2 | -554.85 | 49.2853 | -60.15 | 135.4 | 2.747267 |
| 4 | 2 | -479.6 | 19.9404 | 75.25 | 48.3 | 2.422217 |
| 5 | 2 | -452.65 | 4.7376 | 26.95 | 10.4 | 2.195197 |
| 6 | 2 | -436.1 | 1.9799 | 16.55 | 14.05 | 7.096322 |
| 7 | 2 | -433.6 | 1.8385 | 2.5 | 5.9 | 3.209177 |
| 8 | 2 | -437 | 0.2828 | -3.4 | 1.8 | 6.363961 |
| 9 | 2 | -438.6 | 2.4042 | -1.6 | 1.8 | 0.748701 |
| 10 | 2 | -438.4 | 1.8385 | 0.2 | NA | NA |
|  |  |  |  |  |  |  |

Table S8. Mode and quantiles of the posterior distributions of the estimated demographic parameters for the *Eurycea lucifuga* microsatellite dataset using DIYABC. Years since divergence are estimated by applying age at sexual maturity (Petranka, 1998) to the mean estimates of t1, t2, and t3.

| **Parameter** | **mean** | **median** | **mode** | **q .025** | **q .975** | **Years since divergence** | |
| --- | --- | --- | --- | --- | --- | --- | --- |
| N1 (North-central) | 2.32E+03 | 2.26E+03 | 2.15E+03 | 3.69E+03 | 4.00E+03 |  |  |
| N2 (South-central) | 2.25E+03 | 1.68E+03 | 1.05E+03 | 3.52E+02 | 7.62E+03 |  |  |
| N3 (Eastern) | 8.09E+02 | 5.32E+02 | 3.86E+02 | 1.22E+02 | 3.75E+03 |  |  |
| N4 (Western) | 3.93E+02 | 2.56E+02 | 1.84E+02 | 6.48E+01 | 1.81E+03 |  |  |
| t1 | 7.09E+02 | 6.27E+02 | 5.23E+02 | 1.21E+02 | 1.77E+03 | 1,772.5-2,836 |  |
| t2 | 1.17E+03 | 1.00E+03 | 7.07E+02 | 3.10E+02 | 3.04E+03 | 2,925-4,680 |  |
| t3 | 3.86E+03 | 3.45E+03 | 2.76E+03 | 1.01E+03 | 8.83E+03 | 9,650-15,440 |  |
| µmic_1 | 1.22E-04 | 1.13E-04 | 1.00E-04 | 1.00E-04 | 1.96E-04 |  |  |
| pmic_1 | 2.28E-01 | 2.32E-01 | 2.53E-01 | 1.36E-01 | 2.97E-01 |  |  |
| snimic_1 | 4.91E-08 | 1.71E-08 | 1.00E-08 | 1.00E-08 | 2.81E-07 |  |  |
